# A green Heck reaction protocol towards trisubstituted alkenes, versatile pharmaceutical intermediates

**DOI:** 10.3389/fchem.2024.1431382

**Published:** 2024-07-10

**Authors:** Giacomo Rossino, Giorgio Marrubini, Margherita Brindisi, Marc Granje, Pasquale Linciano, Daniela Rossi, Simona Collina

**Affiliations:** ^1^ Department of Drug Sciences, University of Pavia, Pavia, Italy; ^2^ Department of Pharmacy, University of Naples “Federico II”, Naples, Italy; ^3^ Faculty of Pharmacy and Food Sciences, University of Barcelona, Barcelona, Spain

**Keywords:** Heck reaction, trisubstituted alkenes, green solvents, microwaves, catalysis, design of experiments, DOE

## Abstract

The Heck reaction is widely employed to build a variety of biologically relevant scaffolds and has been successfully implemented in the production of active pharmaceutical ingredients (APIs). Typically, the reaction with terminal alkenes gives high yields and stereoselectivity toward the *trans-*substituted alkenes product, and many green variants of the original protocol have been developed for such substrates. However, these methodologies may not be applied with the same efficiency to reactions with challenging substrates, such as internal olefins, providing trisubstituted alkenes. In the present work, we have implemented a Heck reaction protocol under green conditions to access trisubstituted alkenes as final products or key intermediates of pharmaceutical interest. A set of preliminary experiments performed on a model reaction led to selecting a simple and green setup based on a design of experiments (DoE) study. In such a way, the best experimental conditions (catalyst loading, equivalents of alkene, base and tetraalkylammonium salt, composition, and amount of solvent) have been identified. Then, a second set of experiments were performed, bringing the reaction to completion and considering additional factors. The protocol thus defined involves using EtOH as the solvent, microwave (mw) irradiation to achieve short reaction times, and the supported catalyst Pd EnCat^®^40, which affords an easier recovery and reuse. These conditions were tested on different aryl bromides and internal olefines to evaluate the substrate scope. Furthermore, with the aim to limit as much as possible the production of waste, a simple isomerization procedure was developed to convert the isomeric byproducts into the desired conjugated *E* alkene, which is also the thermodynamically favoured product. The approach herein disclosed represents a green, efficient, and easy-to-use handle towards different trisubstituted alkenes via the Heck reaction.

## 1 Introduction

**FIGURE 1:**
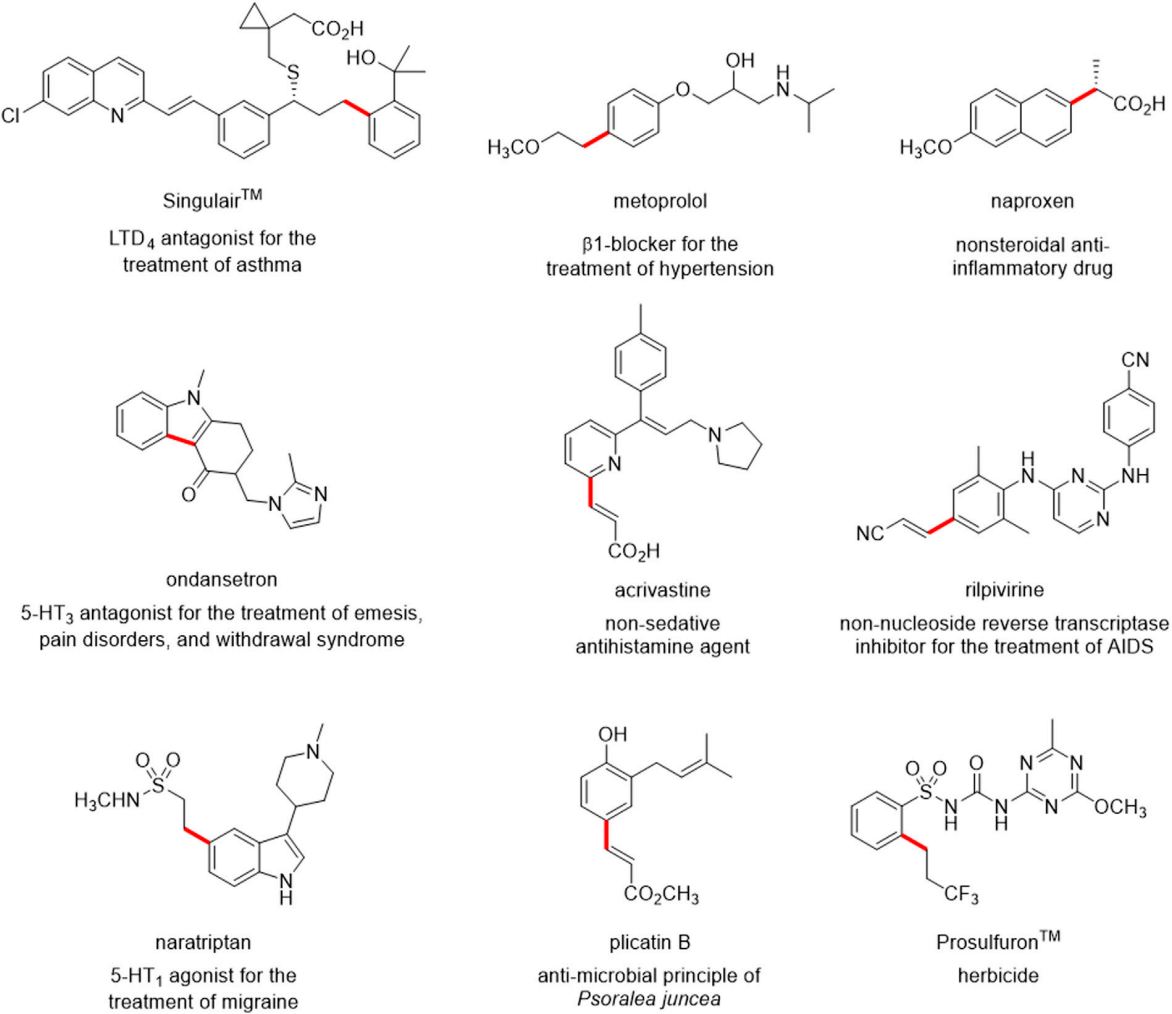
Examples of biologically active molecules synthesized using the Heck reaction. The bond highlighted in red is formed in the cross-coupling Heck reaction.

The Mizoroki-Heck reaction (often referred to simply as Heck reaction) is one of the most popular Pd-catalysed cross-coupling reactions, allowing easy access to substituted alkenes (Oestreich, 2009; Jagtap, 2017). This reaction is now an indispensable implement in the organic chemist’s toolbox, and it has been used in synthesizing a variety of active pharmaceutical ingredients (APIs), clinical candidates, natural products and fine chemicals (Prashad, 2004; Bollikonda et al., 2015; Neumann et al., 2020; Zhang, 2021; Deng et al., 2022; Tabassum et al., 2022; Cai et al., 2023; Stanway-Gordon et al., 2023; Moschona et al., 2024). Some notable examples are reported in Figure 1.

**FIGURE 2:**
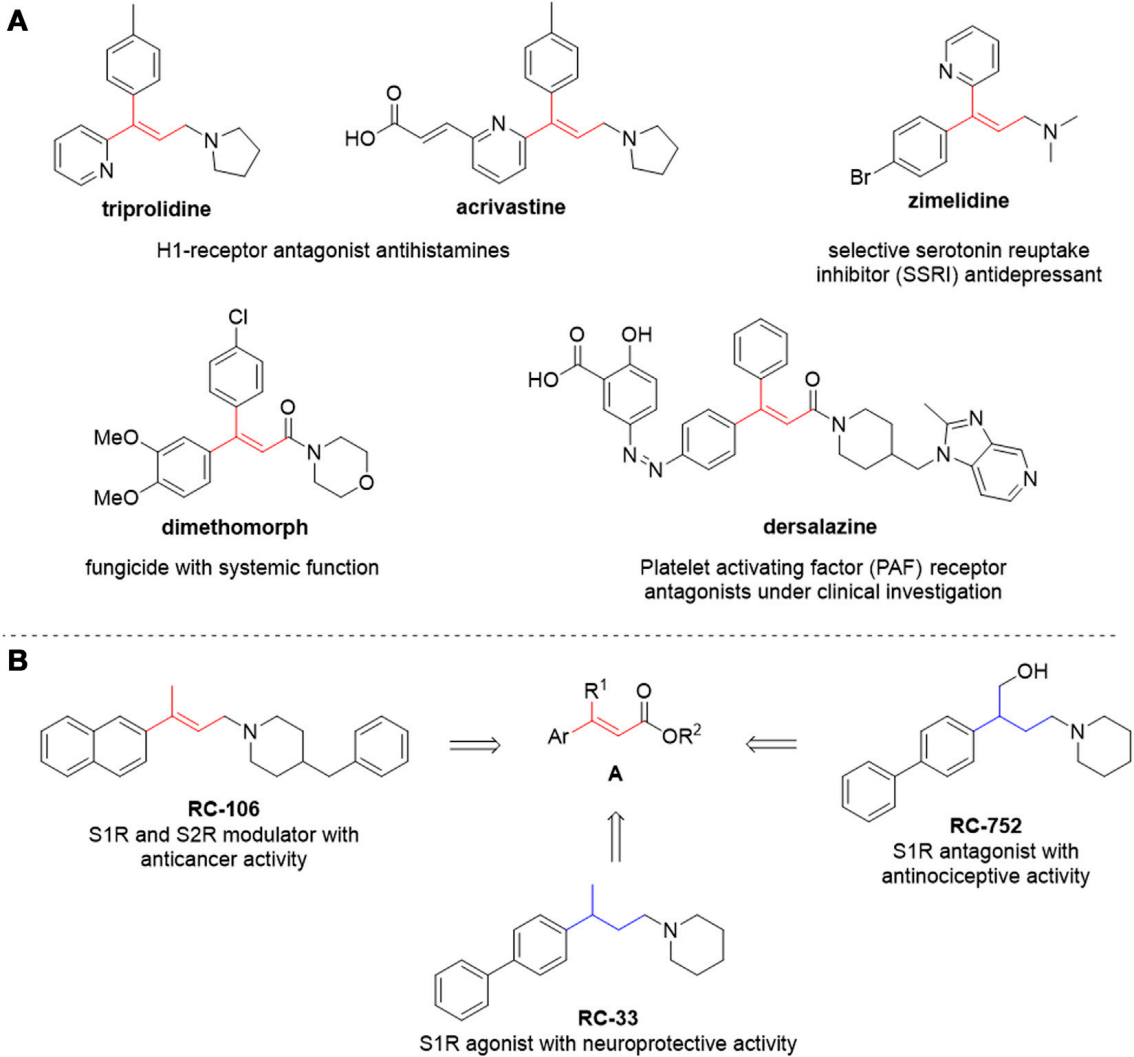
**(A)** compounds of pharmaceutical interest with a trisubstituted alkene; **(B)** lead compounds under investigation in our research group and the general structure of their synthetic precursors bearing a trisubstituted alkene moiety.

Since the original works by Mizoroki and Heck in the 1970s (Mizoroki et al., 1971; Heck and Nolley, 1972; Dieck and Heck, 1974), several groups have improved the efficiency, broadened the substrate scope and, more recently, enhanced the greenness and sustainability of this reaction. Particular attention is given to the use of safer solvents, to the recovery and recycling of the catalyst and/or to the use of non-precious metal catalysts, to the use of more efficient energy transfer systems and shorter reaction times while diminishing byproducts and waste (Li et al., 2005; Loddo et al., 2006; Mital et al., 2006; Du and Wang, 2007; Tao et al., 2011; Yuan and Guo, 2012; Parker et al., 2014; Lv et al., 2019; Yousaf et al., 2020; Dohendou et al., 2021, 2023; Stini et al., 2023). However, applying greener protocols to the synthesis of trisubstituted alkenes via Heck reaction is seldom considered, as internal olefins are challenging substrates even under more “traditional,” non-eco-friendly conditions. Such reactions typically involve problematic solvents (such as DMF), longer reaction times, harsher conditions, tailored catalysts and/or specific substrates (e.g., intramolecular reactions) (Gürtler and Buchwald, 1999; Kondolff et al., 2003; Loddo et al., 2006; Nakashima et al., 2020). Furthermore, the *E/Z* selectivity can be lower than the one observed with terminal alkenes, and several byproducts can be obtained, as a consequence of C=C double bond migration and isomerization. Nevertheless, trisubstituted alkenes as such are embedded in the structure of several drugs, drug candidates, and pesticides (Figure 2A) or are useful intermediates for further modifications (e.g., stereoselective reduction) to provide biologically active compounds. Conventional synthetic methods are generally used in drug discovery, and large amounts of waste are still generated daily in research laboratories. From an environmental and ecological perspective, it is relevant to replace traditional chemical protocols with benign alternatives and incorporating environmentally friendly strategies into the available synthetic portfolio of modern drugs. In this context, developing a green and efficient synthetic strategy to obtain trisubstituted alkenes is of relevant interest.

**SCHEME 1:**
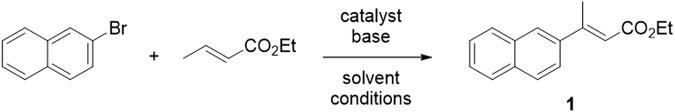
General scheme of the model Heck reaction selected for the present work. The aryl bromide is the limiting reagent. Different catalysts, bases, solvents, and other reaction conditions have been explored, as reported in Table 1.

The versatility of trisubstituted alkenes as final scaffolds and useful synthetic intermediates is well exemplified by compounds with general structure **A** (Figure 2B). These structural templates are involved in several medicinal chemistry projects ongoing in our research group, whose interests include the identification of novel neuroprotective and anticancer agents (Listro et al., 2020, 2022; Rossino et al., 2021; Collina et al., 2023; Linciano et al., 2023). Our most advanced compounds are shown in Figure 2B: these are modulators of the Sigma receptors (SRs) whose selectivity over receptor subtypes and pharmacological activity can be tweaked by small structural changes. On the one hand, the double bond is essential for interacting with Sigma-1 and Sigma-2 receptors (S1R and S2R, respectively) and to exert cytotoxic and pro-apoptotic effects. RC-106, our in-house developed pan-sigma modulator, has promising anticancer activity (Tesei et al., 2019; Cortesi et al., 2020). On the other hand, the double bond can be reduced to switch to a selective S1R modulation, thus promoting neuroprotective or antinociceptive effects (as in the case of RC-33 and RC-752, respectively) (Marra et al., 2016; Rossino et al., 2023). Therefore, we focused on greening the synthesis of compounds with general scaffold **A**, using the synthesis of *E*-**1** (Scheme 1) as a model reaction. *E*-**1** is the precursor of RC-106, currently undergoing further biological investigations to assess its viability as a chemotherapeutic agent.

From an environmental and ecological perspective, this work focuses on developing an efficient, scalable, and sustainable synthesis of trisubstituted alkenes. Different Heck reaction protocols were screened for the identification of suitable reaction conditions. Then, a design of experiments (DoE) approach allowed us to identify the impact of different factors on the reaction yield. The possibility of recycling the byproducts by isomerization was also investigated, and finally, the substrate scope was assessed.

## 2 Materials and methods

Reagents and solvents for synthesis and HPLC analysis were purchased from Sigma Aldrich, Carlo Erba Reagenti, and VWR. HPLC grade water was provided by Q-POD^®^ Milli-Q, endowed with Vent Filter MPK01, “Elix Technology Inside”.

TLC Silica Ge l 60 F_254_ from Merck (Darmstadt, Germany) was used for thin-layer chromatography (TLC). The UV detector MinUVIS DESAGA^®^ Sastedt-GRUPPE equipped with an ultraviolet radiation lamp (λ = 254 and 366 nm) was used for TLC analysis. Silica gel (60 Å, 230–400 Mesh-Sigma Aldrich, Milano, Italy) was used for flash chromatography. Solvents were evaporated under reduced pressure with the Heidolph Laborota 4000 Efficient rotary evaporator (Heidolph Instruments GmbH & Co. KG, Schwabach, Germany).

The mono mode oven Discover^®^ SP instrument (CEM Corporation, Matthews, NC, United States) performed reactions under microwave irradiations. Reactions in an anhydrous and inert environment were carried out in glassware dried with a heat gun and conditioned with N_2_. Plastic syringes conditioned with N_2_ were used to transfer anhydrous solvents and reagents. An analytical balance E42-B (Gibertini, Novate Milanese, Italy) of flow 120 g and sensitivity 0.0001 g was used for all weighings.

NMR spectra were registered at 400 MHz, with instrument ADVANCE 400 MHz (Bruker, Germany) using BBI 5 mm Probe and tetramethyl silane (TMS) as the internal standard (δ = 0.00 ppm). The samples were recorded at room temperature after solubilization in deuterated solvents. TopSpin, iNMR, and MestReNova software were used for spectra recording and elaboration. Chemical shifts are expressed in parts per million (ppm). The following abbreviations are used for multiplicities: s (singlet), d (doublet), dd (doublet of doublet), t (triplet), q (quartet) and m (multiplet).

HPLC analyses of *E*-**1** crudes (DoE study and subsequent experiments) were performed with a JASCO bench-top HPLC system equipped with a quaternary pump model PU-4180, autosampler model AS-4050 with a 5 μL loop, column thermostatic compartment model CO-4060, and UV-Vis variable wavelength detector model UV-4070 with a cell volume of 17 µL (JASCO 2023). The column Merck Purospher®STAR RP-18 end-capped 3 μm (100 mm × 2.1 mm) was used. An isocratic elution was carried out with the following method (Method 1):

- Flux: 0.1 mL/min;
- Injection volume: 1 μL;
- Detection wavelength (λ): 230 nm
- Mobile phase: H_2_O:ACN 25:75 with 0.1% CH_2_O_2_

HPLC analyses for substrate scope assessment were performed with a JASCO bench-top HPLC system, a pump model PU-4185-Binary, autosampler AS-2055 Plus, multiwavelength detector MD-2010 Plus, and interface box LC-Net II/ADC. The column Merck Purospher®STAR RP-18 end-capped 5 μm (150 mm × 4.6 mm) was used. Each crude was analyzed with one of the gradient methods reported in Supplementary Material (Method 2–6, Supplementary Tables S1–S5), using ACN/H_2_O + 0.1% CH_2_O_2_ as mobile phase (solvent A: H_2_O + 0.1% CH_2_O_2_, solvent B: ACN + 0.1% CH_2_O_2_); V_inj_: 1 μL; flow: 1 mL/min; λ = ideally selected for each chromatogram. HPLC systems were operated by the JASCO ChromNAV software (version 2.04.03), which runs in a Microsoft Windows 7 Professional environment.

### 2.1 Synthesis of E-**1** via conventional Heck reaction protocol

The following protocol describe the reaction reported in Table 1, entry 1. Solid reagents were added in an anhydrous two-necked round bottom flask of 25 mL under N_2_ atmosphere: 100 mg of 2-bromonaphthalene (0.48 mmol, 1 equiv.) 160 mg of Et_4_NCl (0.97 mmol, 2 equiv.), 80 mg of AcONa (0.97 mmol, 2 eq) and 12 mg of Pd EnCat^®^40 (loading 0.4 mmol/g, 0.005 mmol, 1 mol%). Reagents were solubilized in 10 mL of DMF, and then 90 µL (0.72 mmol, 1.5 equiv.) of ethyl crotonate were added. The reaction mixture was stirred under heating (oil bath) at 105°C and monitored by TLC (hexane/AcOEt 90:10 v/v). After 5.5 h, the TLC revealed complete consumption of the 2-bromonaphthalene. The reaction mixture was cooled to r.t., filtered on a pleated paper filter into a separatory funnel, diluted with AcOEt (25 mL), and washed with brine (6 × 25 mL). The aqueous phase was extracted with fresh AcOEt (150 mL). The reunited organic phases were dried over anhydrous Na_2_SO_4_, filtered on paper, and concentrated under reduced pressure. The crude obtained (124 g) was purified by flash chromatography, and the product *E*-**1** was isolated as a white solid (59 mg) in 51% yield.

**TABLE 1:** Preliminary tests on Heck reaction conditions.

Entry	Catalyst	Base	Additive	Solvent	Heating	T (°C)	Time (min)	Yield (%)	Ref.
1	Pd EnCat^®^40	AcONa	Et_4_NCl	DMF	oil bath	105	300	51	Listro et al. (2020), Rossino et al. (2023)
2	Pd EnCat^®^40	AcONa	Et_4_NCl	DMF	mw	105	30	50	Loddo et al. (2006)
3	Pd EnCat^®^40	AcONa	Et_4_NCl	Cyrene™	mw	120	30	n.d.	Stini et al., (2023)
4	Pd EnCat^®^40	AcONa	Et_4_NCl	ethylene carbonate	mw	120	30	n.d.	Parker et al. (2014)
5	CuI	K_2_CO_3_	DABCO	EtOH	oil bath	80	360	n.d.	Li et al. (2005)
6	CuI	K_2_CO_3_	DABCO	EtOH	mw	100	30	n.d.	
7	Pd EnCat^®^40	Et_3_N	Bu_4_NBr	[bmim]PF_6_	oil bath	70	240	n.d.	Mital et al. (2006)
8	Pd EnCat^®^40	Et_3_N	Bu_4_NBr	[bmim]PF_6_	mw	100	30	n.d.	
9	Pd EnCat^®^40	K_2_HPO_4_		EtOH/H_2_O 3:2	mw	100	30	n.d.	Yuan and Guo (2012)
10	Pd EnCat^®^40	K_2_HPO_4_		EtOH/H_2_O 3:2	mw	100^a^	30	n.d.	
11	Pd EnCat^®^40	K_2_HPO_4_		EtOH/H_2_O 4:1	mw	110^a^	30	8	
12^b^	Pd EnCat^®^40	AcONa	Et_4_NCl	EtOH/H_2_O 4:1	mw	110	30	15	—

n.d., not detected; DABCO, diazabicyclooctane; [bmim]PF_6_, 1-Butyl-3-methylimidazolium hexafluorophosphate.

^a^ mw irradiation power is set to 200W.

^b^ Pd EnCat^®^40 (3 mol%), ethyl crotonate (1.2 eq), AcONa (1.4 eq), Et_4_NCl (1.2 eq).

### 2.2 General method for the synthesis of E-**1** according to the DoE

Solid reagents were added in a 10 mL vial for mw oven: 2-bromonaphthalene (100 mg, 0.48 mmol 1 equiv.), Et_4_NCl (50–250 mg, 0.6–3 equiv.), AcONa (50–100 mg, 1.3–2.5 equiv.) and Pd EnCat^®^40 (10–20 mg, 0.8–1.6 mol%). The reagents were dispersed in an EtOH/H_2_O mixture (ratio 1:9–9:1, volume 2–5 mL), and then ethyl crotonate (60–100 μL, 1–1.7 equiv.) was added. The reaction mixture was heated by microwave radiation at 120°C (200 W) for 3 cycles of 10 min each (ramp time 1 min) under high magnetic stirring. The reaction was monitored by TLC (hexane/EtOAc 90:10 v/v). The mixture was then filtered using a paper filter and concentrated under reduced pressure. The residue was solubilized in AcOEt (15 mL) and washed with water (3 × 15 mL). The aqueous phase was extracted with fresh AcOEt (45 mL). The reunited organic phases were dried over anhydrous Na_2_SO_4_, filtered, and concentrated under reduced pressure. The crudes obtained following the general procedure were weighted and analyzed using HPLC Method 1. The yields were calculated according to the formula reported in Results and Discussion.

### 2.3 Gram-scale synthesis of E-**1** via green Heck reaction protocol

The following protocol describe the reaction reported in Table 3, Exp 14. Solid reagents were added in a 30 mL vial for mw oven: 2-bromonaphthalene (1 g, 4.8 mmol, 1 equiv.), Et_4_NCl (2.5 g, 3 equiv.) AcONa (1 g, 2.5 equiv.) and Pd EnCat^®^40 (100 mg, 0.8 mol%). Reagents were dispersed in ethanol (15 mL) and then ethyl crotonate (0.6 mL, 1 equiv.) was added. The reaction mixture was heated by mw radiation at T = 140°C (200 W) for a single cycle of 30 min (ramp time 1 min) under high magnetic stirring. The reaction mixture was then filtered using a pleated paper filter, washed with EtOH (20 mL), and dried under reduced pressure. The EtOH was recovered after distillation. The residue was solubilized in AcOEt (40 mL) and washed with water (3 × 40 mL). The aqueous phase was extracted with fresh AcOEt (50 mL). The reunited organic phases were dried with anhydrous Na_2_SO_4_, filtered, and concentrated under reduced pressure. The crude (1.20 g) was purified by flash chromatography (gradient from hexane/AcOEt 95:5 v/v to hexane/AcOEt 90:10 v/v). Product *E*-**1** was isolated as a white solid (578 mg) in 50% yield.

*Ethyl (E)-3-(naphthalen-2-yl)but-2enoate (E-* ***1*** *).* R_f_: 0.32 (hexane/AcOEt 95:5 v/v). ^1^H NMR (400 MHz, CDCl_3_) δ 7.86 (s, 1H, Ar), 7.80–7.71 (m, 3H, Ar), 7.51 (d, *J* = 8.6 Hz, 1H, Ar), 7.44–7.38 (m, 2H, Ar), 6.20 (s, 1H, CH_3_C = C*H*), 4.16 (q, *J* = 7.1 Hz, 2H, OC*H*_*2*_CH_3_), 2.60 (s, 3H, C*H*_*3*_C = CH), 1.25 (t, *J* = 7.1 Hz, 3H, OCH_2_C*H*_*3*_). ^13^C NMR (101 MHz, CDCl_3_) δ 165.86, 154.19, 138.33, 132.45, 132.09, 127.46, 127.10, 126.54, 125.64, 125.45, 124.89, 122.92, 116.49, 58.86, 16.87, 13.34. HPLC (Method 1): tR = 6.67.

### 2.4 Pd EnCat^®^ 40 recycling procedure

PdEnCat^®^40 beads were recovered from the filter paper and washed on Buckner under reduced pressure. Wash cycles with water and *i*-PrOH were carried out to remove impurities. A final wash with EtOH was useful for reconditioning the catalyst. The catalyst thus recovered was used in a second run under the conditions of Exp #14 (Table 3), and the product *E*-**1** was obtained in comparable yield (48%).

### 2.5 Synthesis of E-**1** via isomerization reaction

340 mg of the mixture containing *Z*-**1** and **3** were solubilized in 15 mL of *i*-PrOH in a 30 mL mw vial. Catalytic iodine (one granule) was added. The reaction mixture was heated by mw radiation at T = 120°C (200 W) for a single cycle of 20 min (ramp time 1 min) under high magnetic stirring. Afterward, the reaction mixture was dried under reduced pressure, then solubilized in EtOAc and transferred into a separatory funnel. The organic phase was washed with Na_2_S_2_O_5_ aqueous solution (1 × 50 ml) and brine (1 × 50 ml), then dried with Na_2_SO_4_, filtered, and concentrated under reduced pressure. The crude was purified by flash chromatography (hexane/AcOEt 95:5 v/v). 160 mg of pure *E*-**1** isomer were isolated (47% yield).

### 2.6 General method for the synthesis of **6**–**29** via green Heck reaction protocol (substrate scope assessment)

Solid reagents were added in a 10 mL vial for mw oven: aryl bromide (100 mg, 1 equiv.), Et_4_NCl (3 equiv.) AcONa (2.5 equiv.) and Pd EnCat^®^40 (0.8 mol%). Reagents were dispersed in ethanol (2 mL), and then the alkene (1 equiv.) was added (either purchased or prepared as reported in the Supplementary Material). The reaction mixture was heated by mw radiation at T = 140°C (200 W) for a single cycle of 30 min (ramp time 1 min) under high magnetic stirring. The reaction mixture was filtered using a pleated paper filter and dried under reduced pressure. The residue was solubilized in AcOEt and washed with water. The aqueous phase was extracted with fresh AcOEt. The reunited organic phases were dried over anhydrous Na_2_SO_4_, filtered, and concentrated under reduced pressure. The crudes obtained following the general procedure were weighted and analyzed by NMR and HPLC (Method 2–6, Supplementary Tables S1–S5). Some representative ^1^H-NMR spectra are reported in the Supplementary Material. The yields were calculated according to the formula reported in Results and Discussion.

*Ethyl (E)-3-phenylbut-2-enoate (* ***6*** *).* Colorless oil. Yield: 46%. R_f_: 0.47 (hexane/AcOEt 95:5 v/v). ^1^H-NMR consistent with literature (Yoo et al., 2006; Pugliese et al., 2023) (see Supplementary Material). HPLC (Method 2): tR = 17.6.

*Ethyl (E)-3-(4-methoxyphenyl)but-2-enoate (* ***7*** *).* Pale yellow oil. Yield: 35%. R_f_: 0.48 (hexane/AcOEt 90:10 v/v). ^1^H-NMR consistent with literature (Hofmayer et al., 2019; Pugliese et al., 2023) (see Supplementary Material). HPLC (Method 2): tR = 17.4.

*Ethyl (E)-3-(4-hydroxyphenyl)but-2-enoate (* ***8*** *).* Off-white solid, m.p. 91°C–93°C. Yield: 27%. R_f_: 0.31 (hexane/AcOEt 80:20 v/v + 0.1% HCO_2_H). ^1^H-NMR consistent with literature (Littke and Fu, 2001; Zimmermann et al., 2019) (see Supplementary Material). HPLC (Method 4): tR = 14.6.

*Ethyl (E)-3-(4-(trifluoromethyl)phenyl)but-2-enoate (* ***9*** *).* Colorless oil. Yield: 76%. R_f_: 0.47 (hexane/AcOEt 95:5 v/v). ^1^H-NMR consistent with literature (Pugliese et al., 2023) (see Supplementary Material). HPLC (Method 3): tR = 13.9.

*Ethyl (E)-3-(4-nitrophenyl)but-2-enoate (* ***10*** *).* Waxy solid. Yield: 51%. R_f_: 0.33 (hexane/AcOEt 90:10 v/v). ^1^H-NMR consistent with literature (De Vries, 2018; Paparella et al., 2024) (see Supplementary Material). HPLC (Method 2): tR = 17.0.

*Ethyl (E)-3-([1,1′-biphenyl]-4-yl)but-2-enoate (* ***11*** *).* White solid, m.p. 82°C–84 °C. Yield: 63%. R_f_: 0.48 (hexane/AcOEt 90:10 v/v). ^1^H-NMR consistent with literature (Rossi et al., 2013; Marra et al., 2016) (see Supplementary Material). HPLC (Method 3): tR = 17.0.

*Ethyl (E)-3-(6-methoxynaphthalen-2-yl)but-2-enoate (* ***12*** *)*. Waxy solid. Yield: 58%. R_f_ 0.45 (hexane/AcOEt 95:5 v/v). ^1^H-NMR (400 MHz, CDCl_3_): δ 7.80 (s, 1H, Ar), 7.65 (dd, *J* = 14.9, 8.8 Hz, 2H, Ar), 7.50 (dd, *J* = 8.6, 1.8 Hz, 1H, Ar), 7.10–7.04 (m, 2H, Ar), 6.19 (brs, 1H, CH_3_C=C*H*), 4.16 (q, *J* = 7.1 Hz, 2H, OC*H*_*2*_CH_3_), 3.84 (s, 3H, OC*H*_*3*_), 2.50 (d, *J* = 1.1 Hz, 3H, C*H*_*3*_C=CH), 1.25 (t, *J* = 7.1 Hz, 3H, OCH_2_C*H*_*3*_). ^13^C NMR (101 MHz, CDCl_3_) δ 167.04, 158.39, 155.29, 137.05, 134.86, 130.07, 128.56, 126.95, 125.82, 124.46, 119.36, 116.66, 105.59, 59.84, 55.36, 17.78, 14.40.

*Ethyl (E)-3-(6-hydroxynaphthalen-2-yl)but-2-enoate (* ***13*** *).* Pale yellow oil. Yield: 54%. R_f_: 0.46 (hexane/AcOEt 70:30 v/v). ^1^H NMR (400 MHz, CDCl_3_) δ 7.91 (s, 1H, Ar), 7.79 (d, *J* = 8.6 Hz, 1H, Ar), 7.69–7.67 (m, 1H, Ar), 7.60–7.58 (m, 1H, Ar), 7.16–7.14 (m, 2H, Ar), 6.29 (s, 1H, CH_3_C=C*H*), 4.26 (q, *J* = 7.1 Hz, 2H, OC*H*_*2*_CH_3_), 2.70 (s, 3H, C*H*_*3*_C=CH),1.36 (t, *J* = 7.1 Hz, 3H). ^13^C NMR (101 MHz, CDCl_3_) δ 166.71, 156.43, 150.89, 134.97, 134.46, 128.48, 126.96, 126.84, 124.85, 124.79, 119.05, 116.62, 109.46, 59.78, 18.64, 14.22. HPLC (Method 5): tR = 9.5.

*Ethyl (E)-3-(quinolin-3-yl)but-2-enoate (* ***14*** *).* Yellow solid, m.p. 59°C–63°C. Yield: 71%. R_f_: 0.30 (hexane/AcOEt 80:20 v/v). ^1^H-NMR consistent with literature (Elbaum et al., 1995) (see Supplementary Material). HPLC (Method 4): tR = 16.0.

*Ethyl (E)-3-(6-(benzyloxy)naphthalen-2-yl)but-2-enoate (* ***15*** *).* Pale yellow oil. Yield: 57%. R_f_: 0.53 (hexane/AcOEt 80:20 v/v). ^1^H-NMR (400 MHz, DMSO *d*_*6*_): δ 8.11 (d, *J* = 1.6 Hz, 1H, Ar), 7.93 (d, *J* = 9.0 Hz, 1H, Ar), 7.82 (d, *J* = 8.8, 1H, Ar), 7.70 (dd, *J* = 8.7, 1.9 Hz, 1H, Ar), 7.53–7.51 (m, 1H, Ar), 7.46 (brs, 1H, Ar), 7.44–7.40 (m, 2H, Ar), 7.37–7.34 (m, 1H, Ar), 7.27 (dd, J = 8.9, 2.5 Hz, 1H, Ar), 6.31 (brs, 1H, CH_3_C=C*H*), 5.24 (s, 2H, OC*H*_*2*_Ph), 4.16 (q, *J* = 7.1 Hz, 2H, OC*H*_*2*_CH_3_), 2.62 (d, *J* = 1.2 Hz, 3H, C*H*_*3*_C=CH), 1.26 (t, *J* = 7.1 Hz, 3H, OCH_2_C*H*_*3*_). ^13^C NMR (101 MHz, CDCl_3_) δ 167.07, 157.44, 151.14, 136.66, 135.08, 134.40, 128.93, 128.83, 128.68, 128.16, 127.79, 126.83, 125.94, 125.36, 117.04, 114.92, 110.34, 70.20, 59.93, 19.756, 14.65. HPLC (Method 3): tR = 20.1.

*Ethyl (E)-3-(phenanthren-9-yl)but-2-enoate (* ***16*** *).* Colorless oil. Yield: 58%. R_f_: 0.63 (hexane/AcOEt 80:20 v/v). ^1^H-NMR (400 MHz, DMSO *d*_*6*_): δ 8.90 (d, *J* = 8.2, 1H, Ar), 8.84 (d, *J* = 8.2 Hz, 1H, Ar), 8.01 (dd, *J* = 7.73, 1.47, 1H, Ar), 7.88 (dd, *J* = 4.8, 2.3 Hz, 1H, Ar), 7.76–7.66 (m, 5H, Ar), 6.02 (q, *J* = 1.2 Hz, 1H, CH_3_C=C*H*), 4.21 (q, *J* = 7.1 Hz, 2H, OC*H*_*2*_CH_3_), 2.59 (d, *J* = 1.2 Hz, 3H, C*H*_*3*_C=CH), 1.28 (t, *J* = 7.1 Hz, 3H, OCH_2_C*H*_*3*_). ^13^C NMR (101 MHz, CDCl_3_) δ 168.09, 151.64, 135.14, 133.37, 132.47, 132.06, 131.09, 128.75, 128.54, 128.15, 128.13, 127.60, 127.55, 126.73, 125.99, 123.79, 120.67, 60.95, 20.27, 15.43. HPLC (Method 3): tR = 19.2.

*Ethyl (E)-3-(5-acetylthiophen-2-yl)but-2-enoate (* ***17*** *).* Pale yellow oil. Yield: 30%. R_f_: 0.49 (hexane/AcOEt 80:20 v/v). ^1^H-NMR (400 MHz, DMSO *d*_*6*_): δ 7.61 (d, *J* = 4.0 Hz, 1H, Ar), 7.30 (d, *J* = 4.0 Hz, 1H, Ar), 6.35 (brs, 1H, CH_3_C=C*H*), 4.23 (q, *J* = 7.1 Hz, 2H, OC*H*_*2*_CH_3_), 2.59 (d, *J* = 1.3 Hz, 3H, C*H*_*3*_C=CH), 2.55 (s, 3H, C*H*_*3*_C=O), 1.31 (t, *J* = 7.1 Hz, 3H, OCH_2_C*H*_*3*_). ^13^C NMR (101 MHz, CDCl_3_) δ 193.09, 167.84, 142.73, 141.52, 141.37, 132.02, 125.76, 117.18, 61.20, 27.46, 19.49, 15.65. HPLC (Method 2): tR = 15.0.

*Ethyl (E)-3-(naphthalen-2-yl)pent-2-enoate (* ***18*** *).*Waxy solid. Yield: 13%. R_f_: 0.55 (hexane/AcOEt 95:5 v/v). ^1^H NMR (400 MHz, MeOD) δ 8.01 (brs, 1H, Ar), 7.94–7.87 (m, 3H, Ar), 7.63–7.61 (dd, *J* = 8.6, 1.6 Hz, 1H, Ar), 7.54–7.52 (m, 2H, Ar), 6.18 (s, 1H, CH_2_C=C*H*), 4.26–4.21 (q, *J* = 7.1 Hz, 2H, OC*H*_*2*_CH_3_), 3.29–3.23 (q, *J* = 7.5 Hz, 2H, CH_3_C*H*_*2*_C=C), 1.336–1.32 (t, *J* = 6.9 Hz, 3H, OCH_2_C*H*_*3*_), 1.14–1.10 (t, *J* = 7.3 Hz, 3H, C*H*_*3*_CH_2_C=C). ^13^C NMR (101 MHz, MeOD) δ 166.52, 162.06, 137.96, 133.63, 133.33, 128.15, 127.96, 127.17, 126.39, 126.17, 125.88, 123.97, 116.45, 59.60, 23.59, 13.24, 12.69. HPLC (Method 6): tR = 24.6.

*Ethyl (E)-3-phenylpent-2-enoate (* ***19*** *).* Pale yellow oil. Yield: 14%. R_f_: 0.64 (hexane/AcOEt 95:5 v/v). ^1^H-NMR consistent with literature (Appella et al., 1999; Pugliese et al., 2023) (see Supplementary Material). HPLC (Method 4): tR = 28.3.

*Ethyl (E)-4-methyl-3-(naphthalen-2-yl)pent-2-enoate (* ***20*** *).* Colorless oil. Yield: 33%. R_f_: 0.32 (hexane/AcOEt 95:5 v/v). ^1^H NMR (400 MHz, CDCl_3_) δ 7.76–7.70 (m, 3H, Ar), 7.59 (s, 1H, Ar), 7.42–7.40 (m, 2H, Ar), 7.28 (dd, *J* = 8.4, 1.6 Hz, 1H, Ar), 5.74 (s, 1H, C=C*H*CO), 4.17–4.07 (m, 3H, OC*H*_*2*_CH_3_ and (CH_3_)_2_C*H*), 1.24 (t, *J* = 7.1 Hz, 3H, OCH_2_C*H*_*3*_), 1.08 (d, *J* = 7.0 Hz, 6H, (C*H*_*3*_)_2_CH). ^13^C NMR (101 MHz, CDCl_3_) δ 167.09, 166.25, 138.49, 132.87, 132.62, 128.09, 127.62, 127.23, 126.55, 126.32, 126.14, 125.94, 118.92, 59.93, 29.83, 21.52, 14.32.

*Ethyl (E)-4-methyl-3-phenylpent-2-enoate (* ***21*** *).* Colorless oil. Yield: 10%. R_f_: 0.52 (hexane/AcOEt 95:5 v/v). ^1^H-NMR consistent with literature (Antonietti et al., 2005) (see Supplementary Material). HPLC (Method 6): tR = 30.6.

*Ethyl (Z)-4,4,4-trifluoro-3-(naphthalen-2-yl)but-2-enoate (* ***22*** *).* Colorless oil. Yield: 14%. R_f_: 0.30 (hexane/AcOEt 98:2 v/v). ^1^H-NMR (400 MHz, CDCl_3_): δ 7.83–7.76 (m, 4H, Ar), 7.47–7.43 (m, 3H, Ar), 6.09 (q, ^4^ *J*_FH_ = 7.8 Hz, 1H, CF_3_C = C*H*), 4.35 (q, *J* = 7.1 Hz, 2H, OC*H*_*2*_CH_3_), 1.31 (t, *J* = 7.1 Hz, 3H, OCH_2_C*H*_*3*_). ^13^C NMR (101 MHz, CDCl_3_) δ 166.32, 143.77 (q, ^3^ *J*_CF_ = 5.6 Hz), 133.92, 133.01, 130.34, 128.98, 128.66, 127.73, 127.52, 127.18, 126.98, 123.15, 122.29 (q, ^1^ *J*_CF_ = 270.5 Hz), 120.95, 118.26, 116.52 (q, ^2^ *J*_CF_ = 35.4 Hz), 62.29, 13.95.

*Ethyl (E)-3-(naphthalen-2-yl)-3-phenylacrylate (* ***25*** *).* White solid, m.p. 83°C–84°C. Yield: 35%. R_f_: 0.36 (hexane/AcOEt 95:5 v/v). ^1^H-NMR consistent with literature (Zhang et al., 2021) (see Supplementary Material).

*Ethyl (E)-3-(4-nitrophenyl)-3-phenylacrylate (* ***26*** *).* Pale yellow oil. Yield: 10%. R_f_: 0.32 (hexane/AcOEt 95:5 v/v). ^1^H-NMR consistent with literature (Calò et al., 2001) (see Supplementary Material).

*Ethyl 3,3-diphenylacrylate (* ***27*** *)*. Colorless oil. Yield: 23%. R_f_: 0.44 (hexane/AcOEt 95:5 v/v). ^1^H-NMR consistent with literature (Kubota et al., 2012) (see Supplementary Material).

*Ethyl (E)-3-(naphthalen-2-yl)-3-(4-nitrophenyl)acrylate (* ***28*** *).* Waxy solid. Yield: 10%. R_f_: 0.29 (hexane/AcOEt 95:5 v/v). ^1^H NMR (400 MHz, CDCl_3_) δ 8.23 (d, *J* = 8.7, 2H, Ar), 7.77–7.75 (m, 2H, Ar), 7.68–7.66 (m, 1H, Ar), 7.49–7.37 (m, 6H, Ar), 6.54 (s, 1H, C=C*H*), 4.02 (q, *J* = 7.1, 2H, OC*H*_*2*_CH_3_), 1.11 (t, *J* = 7.1 Hz, 3H, OCH_2_C*H*_*3*_). ^13^C NMR (101 MHz, CDCl_3_) δ 165.46, 154.33, 147.63, 146.09, 136.43, 133.83, 132.92, 130.15, 128.89, 128.68, 128.61, 127.66, 127.49, 126.90, 124.38, 123.35, 118.83, 60.50, 14.08. HPLC (Method 6): tR = 27.7.

*Methyl (E)-3-(naphthalen-2-yl)acrylate (* ***29*** *).* Off-white solid, m.p. 65°C–68°C. Yield: 70%. R_f_: 0.3 (hexane/AcOEt 95:5 v/v). ^1^H-NMR consistent with literature (Tyagi and Fasan, 2016; Das et al., 2017) (see Supplementary Material).

## 3 Results and discussion

### 3.1 Preliminary experiments

As the first step in our work, we selected a model Heck reaction as our benchmark and the starting point for developing an efficient green protocol. Our choice fell on the reaction between 2-bromonaphthalene and ethyl crotonate (Scheme 1), performed on 100 mg of aryl bromide as a limiting agent. The bench scale allows an easy quantitation of the yield, and the product (the α,β-unsaturated ester *E*-**1**) is a key intermediate in the synthesis of RC-106, our in-house developed pan-sigma modulator (Tesei et al., 2019; Cortesi et al., 2020). Moreover, an analytical standard of the product was already available in-house.

The reaction was initially performed under “conventional” conditions (Table 1, entry 1), following a protocol already reported by us (Listro et al., 2020; Rossino et al., 2023). The catalyst was Pd EnCat^®^ 40 (0.4 mmol/g Pd loading), a commercial catalyst constituted by palladium acetate microencapsulated in a polyurea matrix (Ley et al., 2002; Ramarao et al., 2002; Pd EnCat, 2022). Sodium acetate was employed as the base, and the reaction was performed in the presence of Et_4_NCl, as it is known that tetraalkylammonium salts can facilitate Heck-type reactions, thus avoiding the use of phosphine ligands (Jeffery, 1996; Jagtap, 2017). The main concern of such a protocol was the use of DMF, which entails several problems in terms of safety and environmental impact. DMF is a flammable solvent associated with acute and reproductive toxicity (Globally Harmonized System hazard statements H226, H312, H319, H332, H360). Moreover, its high boiling point and its miscibility with water make workup procedures cumbersome, requiring numerous washings with brine and contributing to increased waste generation. For these reasons, DMF is generally a red-labeled or banned solvent in several pharmaceutical industries, exemplified by Pfizer, GSK, and Sanofi tables on solvent greenness (Byrne et al., 2016; Bryan et al., 2018; Sharma et al., 2020). Therefore, we initially screened a series of different reaction conditions based on the results of literature research on green Heck reactions. The experimental conditions, yields, and references of the original publications are reported in Table 1. Particular attention was devoted not only to using safer solvents but also to the catalyst choice and energy transfer efficiency. In particular, we envisaged the use of heterogeneous catalysis as a valuable approach to facilitate the recovery and reuse of the catalyst, thus limiting the contamination of the product and compensating for the high costs of the noble metal. In the present work, we employed the readily available commercial catalyst Pd EnCat^®^, which promises to meet the abovementioned requirements (Lee et al., 2005; Baxendale et al., 2006; Carpita and Ribecai, 2009). To be thorough, we must point out that even this product can act via a release and re-capture process typical of Pd/C and other solid catalysts (Broadwater and McQuade, 2006; Richardson and Jones, 2006; Pagliaro et al., 2012). Nevertheless, the comparison between Pd EnCat^®^ and homogeneous Pd(OAc)_2_ has already evidenced clear advantages in terms of recyclability and purity of the products (Baxendale et al., 2006; Pagliaro et al., 2012). On the other hand, we also investigated the possibility of replacing the Pd catalyst *tout court* by employing a non-precious or “base” metal catalyst. The latter choice would be more sustainable since base metals are more abundant, cheaper, typically less toxic, and have reduced carbon footprints associated with their extraction. For these reasons, the search for alternatives to precious metal catalysts is gaining increasing importance, although this is often a very challenging task and a relatively young research field (Bryan et al., 2018; Hayler et al., 2019). Based on a previous report on its application in the Heck reaction, CuI was experimented by us as an alternative to Pd (Li et al., 2005).

In addition, reactions have been conducted under microwave (mw) heating. The use of mw irradiation is a quick and efficient system to convey energy to the reaction mixture, suitable for reducing the reaction times, improving the yields, and reducing the overall cost of a synthetic step. Furthermore, mw irradiation can enhance solubility in alternative solvents and/or reactivity of the substrates. For these reasons, mw-assisted organic synthesis is widely considered a greener alternative to conductive heating systems (de la Hoz et al., 2016; Collina and Volpe, 2017; Sharma et al., 2018; Kar et al., 2022). In these preliminary experiments, the amount of catalyst, olefin, base, and additive were 1 mol%, 1.5 equiv., 2 equiv. and 2 equiv. respectively, and the mw oven power was set to 100W unless otherwise specified. The aim was to identify the most promising experimental conditions suitable for further investigation. Thus, different solvents, bases, temperatures, heating systems, and reaction times were tested based on references to literature and our previous experience. Notably, simply substituting DMF with Cyrene™ or ethylene carbonate (entries 3 and 4) did not give the desired product, even increasing the temperature to 120 °C. The reactions with the Cu catalyst in ethanol (entries 5 and 6) and those with the Pd catalyst in the ionic liquid [bmim]PF_6_ (entries 7 and 8) were not successful, neither with conventional heating nor with microwave irradiation. Entries 9–11 are characterized by a biphasic system obtained by adding K_2_HPO_4_ to an EtOH/H_2_O mixture. The inorganic salt functions as both a base and a phase separator, allowing an easy recovery of the products in the organic phase. With this approach, the reaction performed in EtOH/H_2_O 4:1 (v/v) gave the desired product with an 8% yield. These conditions were further modified (entry 12) by increasing the amount of catalyst (from 1 mol% to 3 mol%) and using the same base and additive as in the reactions performed in DMF. These new “hybrid” conditions increased the yield to 15% and were considered a suitable starting point for the subsequent DoE study. The setup of entry 12 presents significant advantages: using a mixture of green solvents, a heterogeneous catalyst, and a quick and efficient heating system. The modest yield was not considered a limitation, as the crude contained a significant amount of unreacted aryl bromide, prompting us to investigate how to bring the reaction to completion.

### 3.2 Design of experiments (DoE) to study the effects of six factors on the reaction yield

Starting from the conditions of entry 12 (Table 1), an experimental design was performed to assess the impact on yield exerted by different experimental conditions, i.e., the amount of catalyst and reagents, the solvent composition, and its volume. Therefore, the response studied was the yield%, and six factors were considered (x1-x6, Table 2) at two levels (higher value and lower value). The collection of the variation range for all the factors defined the experimental domain. The variation of these factors within the investigated ranges is expected to affect the yield. The DoE study aims to verify this hypothesis, rank the factors according to their impact on the yield, and identify which level of each factor increases the yield. An additional factor (called dummy factor and indicated as e1) was used to estimate the random experimental noise that originates from chemical practice, including human action and the instruments used. The dummy factor is a virtual variable (i.e., unrelated to a chemical or physical change in the system) that is not expected to cause an effect on the studied response (the reaction yield). All experiments used 0.48 mmol (100 mg) of 2-bromonaphthalene as the limiting reagent. The Plackett-Burman design was selected for this work, as it is a well-known low-resolution experimental design that allows a screening of several factors to identify those having a greater effect on the response (Plackett and Burman, 1946). This design is particularly efficient and suited for studying any number of factors greater than four, as it gives maximal information from the least experimental runs.

**TABLE 2:** Experiments performed according to the Plackett-Burman design.

	x1	x2	x3	x4	x5	x6	Y
Exp#^a^	Catalyst (mol%)	Ethyl crotonate (mmol)	Et_4_NCl (mmol)	NaOAc (mmol)	Solvent (EtOH:H_2_O, v/v)	Solvent (mL)	Yield^b^ (%)
*1*	1.6	0.82	1.50	0.61	9:1	2	22
*2*	0.8	0.82	1.50	1.22	1:9	5	1
*3*	0.8	0.48	1.50	1.22	9:1	2	33
*4*	1.6	0.48	0.30	1.22	9:1	5	16
*5*	0.8	0.82	0.30	0.61	9:1	5	10
*6*	1.6	0.48	1.50	0.61	1:9	5	1
*7*	1.6	0.82	0.30	1.22	1:9	2	2
*8*	0.8	0.48	0.30	0.61	1:9	2	1
*Center1*	1.2	0.65	0.9	0.92	1:1	3.5	12
*Center2*	1.2	0.65	0.9	0.92	1:1	3.5	12

The microwave oven settings for all experiments were as follows: 3 heating cycles of 10 min each, at 120°C and 200W.

^a^ The ten experiments are listed in standard order but were conducted randomly. The order in which they were executed is reported in the Supplementary Material.

^b^ Determined by HPLC analysis. Conditions: Merck Purospher®STAR RP-18 endcapped 3 μm (100 mm × 2.1 mm) column; isocratic method (H_2_O/CH_3_CN 25:75 with 0.1% HCO_2_H); flux: 0.1 mL/min; λ = 230 nm.

For this reason, it became very popular and largely used in several areas of investigation, including the development of pharmaceutical products, although it allows studying only first-order models with resolution III, which is the least resolution of fractional factorial designs (Plackett and Burman, 1946; Babu and Divakar, 2001; Wang et al., 2007; Galib et al., 2009; Beg, 2021). In detail, the 8-runs Plackett-Burman design was employed, which involves eight experiments to study the effects of seven factors. Two additional experiments for model validation, were performed in the center of the experimental domain. The experimental plan is reported in Table 2, along with the yield% obtained for each experiment.

The same workup procedure was employed for all experiments. All crudes were subjected to HPLC analysis to determine the chromatographic purity of the target compound (%*E*-**1**). This was used to calculate the yields as follows:

$$yield\%=\frac{\%E‐1\times \left(mg\;crude\right)}{{MW}_{product}\times {mmol}_{aryl\;bromide}}=\frac{\%E‐1\times \left(mg\;crude\right)}{240.3\times 0.483}$$

**FIGURE 3:**
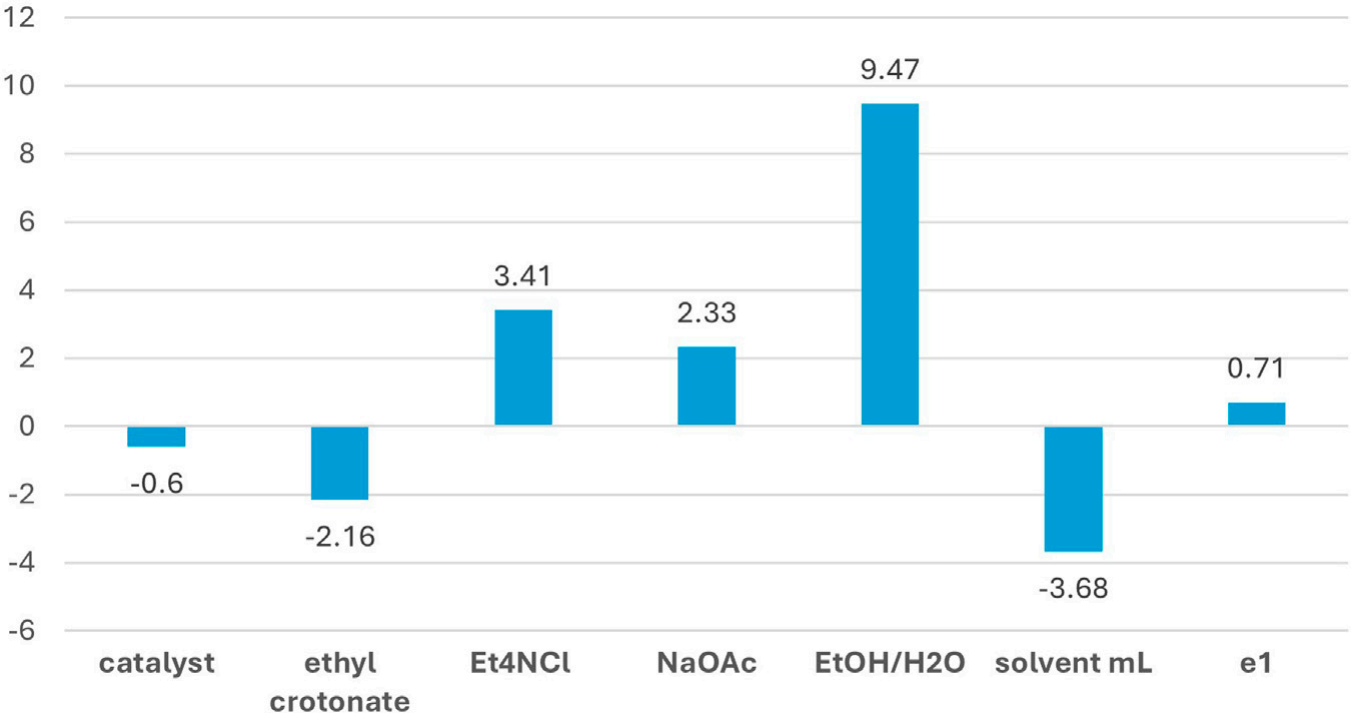
Plot of the regression model coefficients.

All the elaborations reported in this article were performed using the Chemometric Agile Tool (CAT), an R-based software freely downloadable from http://gruppochemiometria.it/index.php/software. The yields were subjected to regression analysis, and the results are reported in Figure 3, which indicates the effective influence of each factor on the studied response (i.e., the reaction yield): the height of the bars is proportional to their incidence on response, while positive or negative sign defines the involved extreme of range (higher value or lower value).

In detail, the EtOH/H_2_O ratio was the most important factor (among those studied in the present DoE) affecting the yield. The positive coefficient indicates that higher EtOH content in the mixture (EtOH/H_2_O = 9:1, v/v) enhances the yield. Such a result is likely due to the higher solubility of the reagents and product in the mixture with a higher percentage of EtOH. The solvent volume also plays an important role, and the negative coefficient reveals improvement in response when the minimum amount of solvent is employed. The same is observed for ethyl crotonate, although with a smaller coefficient. These findings appeal from a green chemistry perspective, as they indicate that the yield can be increased while limiting the amount of solvent and reactant employed, thus saving materials and reducing waste.

On the other hand, the amount of base and tetraalkylammonium salt additive showed a positive influence at their higher level. Therefore, it justifies the excess of Et_4_NCl and AcONa (3 and 2.5 equivalents, respectively) since their effects on the yield are not negligible. In particular, tetraalkylammonium salts promote Heck-type reactions even under phosphine-free conditions, as demonstrated by Jeffery (Jeffery, 1996, 2000). Finally, the amount of the catalyst did not affect the yield (within the experimental domain range of the factors), as its coefficient is lower than that of the dummy factor e1. Thus, the lowest amount of the precious Pd EnCat^®^ catalyst (0.8 mol%) can be used without compromising the yield.

**FIGURE 4:**
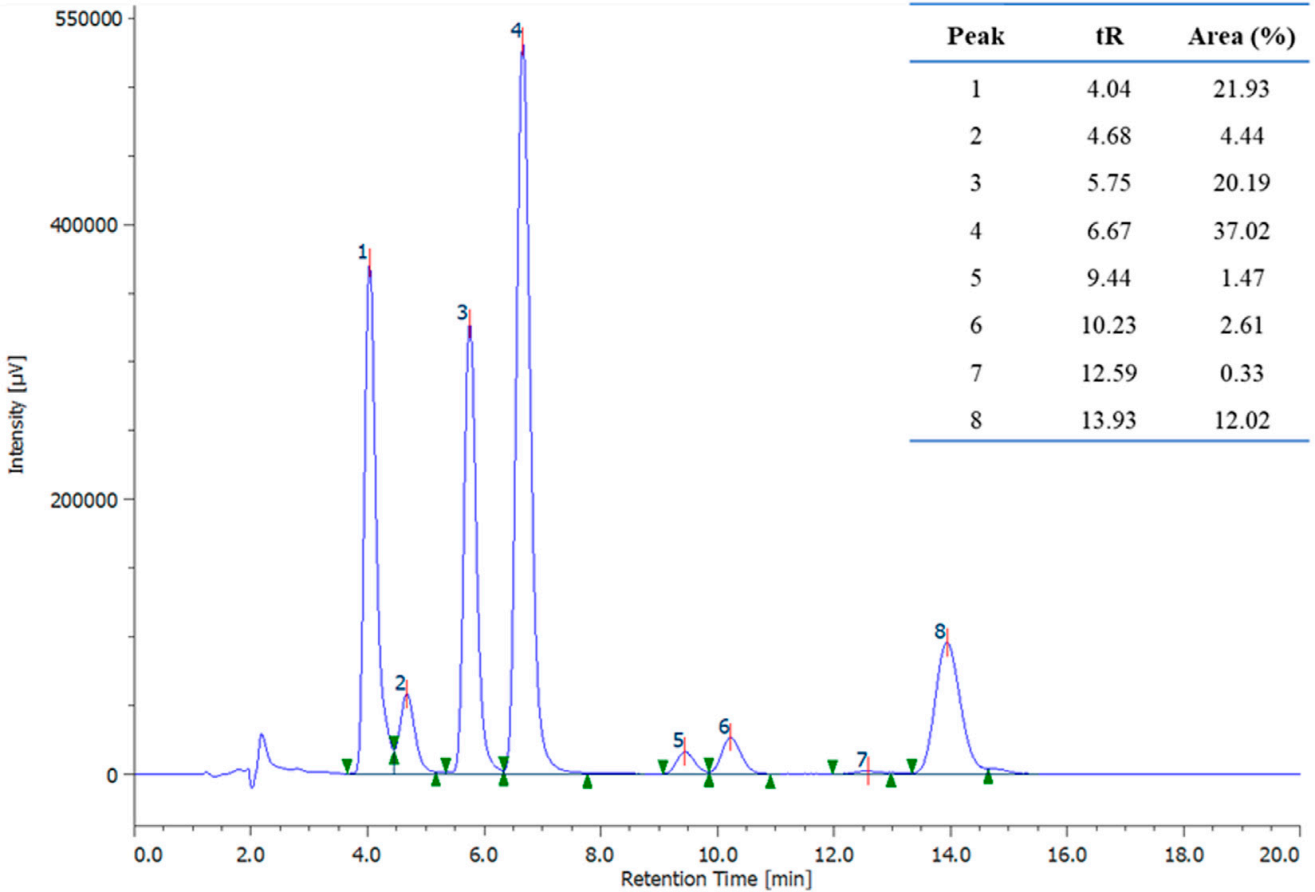
HPLC chromatographic profile of experiment 3 (λ = 230 nm). Peak 3 corresponds to the starting material (2-bromonaphthalene), and peak 4 corresponds to the desired product (*E*-**1**).

The conditions of Exp# 3 (Table 2) are such that each influential factor is set at the level of our experimental space that enhances the studied response. Under these conditions, compound *E*-**1** was obtained in 33% yield, lower than the yield obtained with the reactions performed in DMF. Still, it is also due to incomplete conversion of the starting material (79% conversion), as evidenced by HPLC analysis of the crude (Figure 4). Therefore, the conditions of Exp# 3 were selected for further studies to bring the reaction to completion and increase the yield.

### 3.3 Experiments to increase conversion%

The conditions of Exp# 3 (Table 2) were selected as a new starting point for investigating parameters previously held constant during the DoE study. A series of experiments were conducted, summarized in Table 3, along with the yields obtained.

**TABLE 3:** Final experiments to study the impact of different additives, solvent composition, irradiation cycles, and temperature.

Exp#	Additive	Solvent (EtOH:H_2_O, v/v)	Time (number of cycles x min)	T (°C)	Yield (%)
*9*	Bu_4_NBr	9:1	3 × 10	120	9
*10*	Et_4_NCl	9:1	4 × 10	120	33
*11*	Et_4_NCl	9:1	1 × 30	120	30
*12*	Et_4_NCl	EtOH	1 × 30	120	30^a^
*13*	Et_4_NCl	9:1	1 × 30	140	41^b^
*14*	Et_4_NCl	EtOH	1 × 30	140	51^b^

All reactions were performed on 0.48 mmol (100 mg) of 2-bromonaphthalene.

^a^ A cleaner crude is obtained, with impurities decreasing from 42% to 26%.

^b^ The starting material is completely consumed.

Firstly, Bu_4_NBr was tested as an alternative tetraalkylammonium salt additive (Exp# 9), but this resulted detrimental for the yield. Therefore, Et_4_NCl was maintained for the subsequent experiments. Then, the heating cycles were varied in number and duration. No relevant differences in the yield were observed with 3 or 4 cycles of 10 min each, nor with a single cycle of 30 min. This last setup was selected as the quickest and most efficient. Changing the solvent composition to 100% EtOH did not increase the yield but enhanced the selectivity toward the product of interest. The chromatographic profile of the crude revealed a lower percentage of impurities (26%) than that obtained from Exp# 3 (42%). Such finding also facilitates solvent recycling by simple distillation under reduced pressure, thus diminishing environmental impact. Finally, raising the temperature from 120°C to 140°C resulted in complete consumption of the aryl bromide and an increased yield. The highest yield (51%) was obtained by performing the reaction in pure EtOH at 140°C with a single irradiation cycle of 30 min (Exp #14). These reaction conditions were successfully scaled up to 1g of 2-bromonaphthalene, with the only modification of increasing the concentration from 0.24 M to 0.32 M to avoid an excessive volume of solvent in the 30 mL microwave vial, which might lead to an excessive internal pressure of the instrument. We assumed this would not be detrimental to the reaction since the DoE study evidenced that the minimum level of solvent volume contributes to increasing the yield. The yield calculated on the isolated *E*-**1** product (50%) was superimposable with that calculated for the reaction on a small scale.

### 3.4 Byproducts isolation and recycling

**FIGURE 5:**
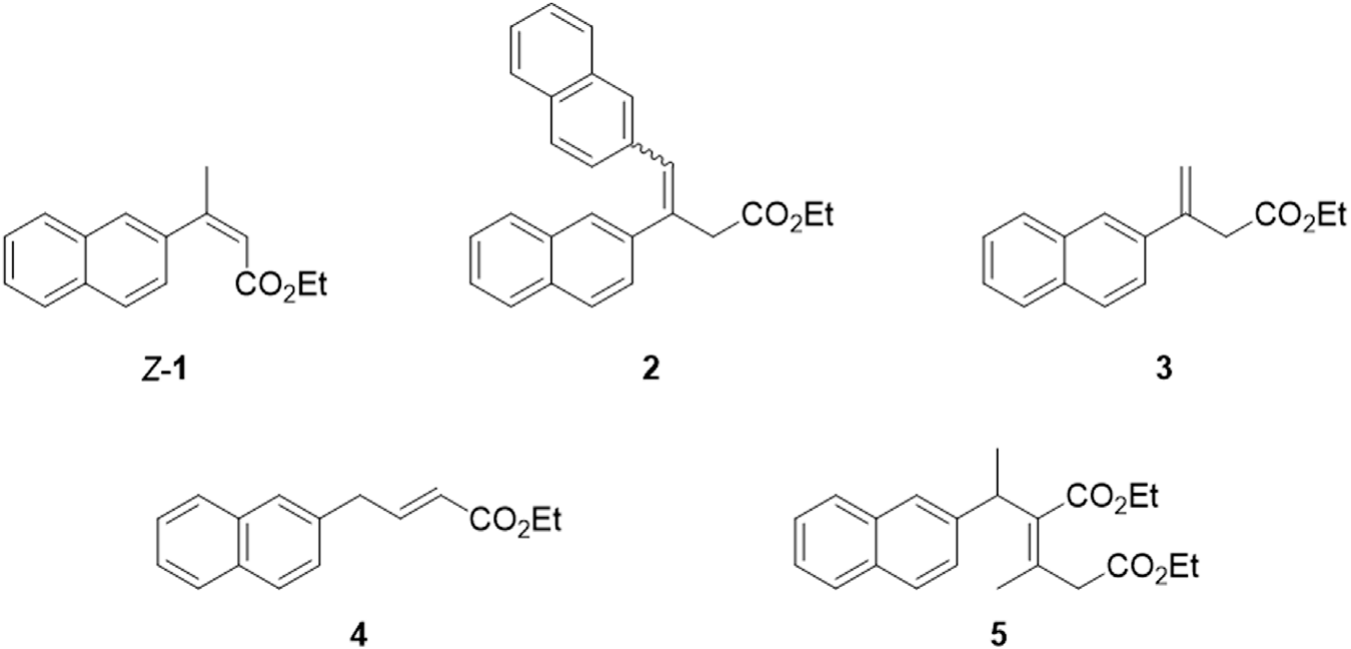
Proposed structures of the main byproducts obtained during the Heck reaction.

The main byproducts were isolated and characterized to evaluate the novel Heck protocol comprehensively. Three fractions, besides the desired *E*-**1**, were isolated through flash chromatography after the gram-scale reaction: fraction **a** (R_f_ = 0.27), fraction **b** (R_f_ = 0.21), and fraction **c** (R_f_ = 0.16) (TLC: n-Hexane/EtOAc 95:5). Each of these fractions was composed by a mixture of two or more byproducts (which structures are reported in Figure 5), as evidenced by spectroscopic and chromatographic data. The main component of fraction **a** was hypothesized to be compound **2**. Fraction **b** resulted in a mixture of isomers of *E*-**1**, including the regioisomer with the terminal alkene (compound **3**), the *Z*-configured alkene (compound *Z*-**1**), and the constitutional isomer **4** (or its regioisomer with the alkene double bond in the β,γ position) in the ratio 1:0.5:0.3. The presence of compound **3** may explain the formation of **2**, assuming a second Heck arylation occurs. These structures were deduced from NMR and MS analyses. Fraction **c** provided a more complex profile at GC-MS: besides three peaks with 240 m/z (isomers of *E*-**1**), a minor peak with 354 m/z was identified, which was correlated to the proposed structure **5** (or one of its regioisomers) that might be formed upon a 1,4 addition of **1** on ethyl crotonate.

To reduce the waste generated in the newly developed Heck protocol, we considered the possibility of recovering the undesired isomers *Z*-**1** and **3**, and converting them into the product of interest *E*-**1**. The conversion can be achieved through an isomerization reaction, under thermodynamic control, that favors the formation of the most stable trisubstituted *E* alkene. Alkenes isomerization has been thoroughly investigated by many research groups using different strategies such as metal catalysis, acid or base catalysis, photocatalysis and radical activation (Yu et al., 2002; Lee and Zaera, 2005; Słomkiewicz, 2006; Canovese et al., 2008; Chahboun et al., 2009; Chahboun et al., 2009; Smarun et al., 2017; Brégent et al., 2020; Neveselý et al., 2022; Wang et al., 2024). Among the many possible approaches to perform this transformation, we selected base or iodine catalysis to prioritize environmentally friendly reagents and avoid using precious metals-based catalysts. Different conditions were experimented with, as reported in Table 4. Since the isolation of each byproduct with high purity is time- and resources-consuming, all reactions were performed on a mixture containing compounds *Z*-**1**, **2,** and **3** (50 mg scale).

**TABLE 4:** Conditions of the isomerization experiments.

Entry	Reagent	Solvent	T (°C)	Heating	Time (min)	Yield^a^ (%)
*1*	*t*-BuOK (2 mol%)	*i*-PrOH	25	—	1,440	8
*2*	*t*-BuOK (4 mol%)	*i*-PrOH	25	—	1,440	n.d.
*3*	*t*-BuOK (0.5 mol%)	*i*-PrOH	25	—	60	11
*4*	*t*-BuOK (0.5 mol%)	*i*-PrOH	25	—	1,440	15
*5*	I_2_ (cat.)^b^	toluene	120	oil bath	900	40
*6*	I_2_ (cat.)	*i*-PrOH	120	mw	10	49
*7*	I_2_ (cat.)	*i*-PrOH	120	mw	20	52
*8*	I_2_ (cat.)	*i*-PrOH	120	mw	30	50

^a^ Determined by HPLC analysis. Conditions: Merck Purospher®STAR RP-18 endcapped 3 μm (100 mm × 2.1 mm) column; gradient (solvent A: H_2_O + 0.1% HCO_2_H; solvent B: CH_3_CN + 0.1% HCO_2_H), λ = 254 nm (see Supplementary Material).

^b^ One bead of molecular iodine was used for each I_2_-catalyzed reaction.

The use of catalytic *t*-BuOK in *i*-PrOH is reported in the literature on a substrate similar to *Z*-**1** (Chen et al., 2014). Despite the long reaction time, the alcohol solvent and the mild conditions were considered amenable from a green chemistry perspective. Therefore, the same conditions were applied to our substrate (entry 1, Table 4). However, only modest results were obtained, even with different equivalents of the catalyst and different reaction times. On the other hand, an iodine-catalyzed protocol had already been experimented with satisfying yield, considering that the starting mixture also contains side-products that cannot be converted into *E*-**1** (e.g., compound **2**). Catalytic iodine promotes *cis*-*trans* isomerization of alkenes and is a well-established tool in organic synthesis (Hepperle et al., 2005; Yusubov and Zhdankin, 2015; Settle et al., 2018). However, the procedure originally employed by us (entry 5) is not compliant with the principles of green chemistry, as it requires refluxing toluene for several hours. Toluene is restricted by REACH with Regulation (EC) No 1907/2006 due to its high environmental and health toxicity. Instead, using *i*-PrOH under mw irradiation provided considerable advantages: satisfying yields, a green solvent, and an efficient heating system. Entry 7 (Table 4) gave the best yield and reaction time results. Therefore, these conditions were scaled to 340 mg, and the product *E*-**1** was isolated by column chromatography with a comparable yield (47%).

### 3.5 Substrate scope

**FIGURE 6:**
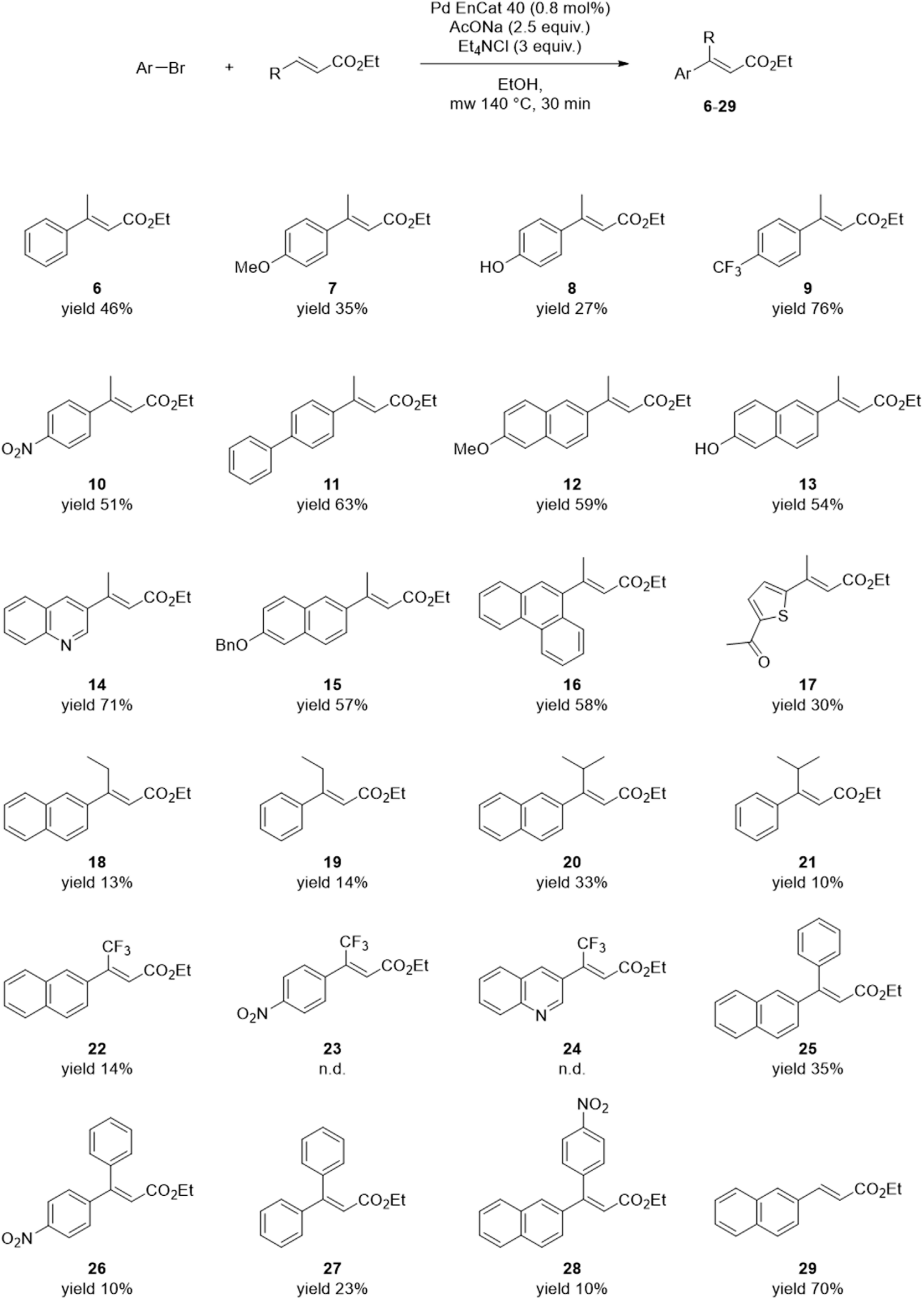
Substrate scope of the developed Heck reaction protocol.

The reaction conditions of the Heck Exp #14 (Table 3) were applied to different aryl bromides and disubstituted alkenes to assess the substrate scope. All reactions were performed on 100 mg of aryl halide. Most of the yields were calculated from the chromatographic purity of the crudes. These were determined by HPLC using slightly different methods, depending on the composition of the mixture. In these cases, either a pure standard of the target product was already available in-house, or the identity of the main product was easily determined by ^1^H-NMR analysis of the crude. When this was not feasible, the crude was purified by flash chromatography. The results are reported in Figure 6.

In the reaction with ethyl crotonate, most aryl bromides gave yields comparable or higher than the model reaction with 2-bromonaphthalene (46%–76% yield). Compound **11** is another product of interest for our medicinal chemistry projects, as it is an intermediate of RC-33 and RC-752, other in-house developed Sigma Receptor modulators. Also, in this case, the yield is superimposable to that obtained in our previous works using DMF, conventional heating, and long reaction times (Rossino et al., 2021, 2023). Exceptions include phenyls with electron-donating groups in the *para* position (i.e., compounds **7** and **8**), probably due to the strengthening effect of the substituent toward the C-Br bond. This hypothesis is consistent with the enhanced reactivity of **9** and **10**, which bear electron-withdrawing groups instead. Different α,β-unsaturated esters, besides ethyl crotonate, were also tested to assess the alkene substrate’s effect. These were purchased or prepared from the commercial carboxylic acid precursor (see Supplementary Material). The α,β-unsaturated esters with longer or branched chains (compounds **18**–**21**) gave lower yields, probably due to the increased steric hindrance of the alkene substrate. Another problematic substrate is the ethyl (*E*)-4,4,4-trifluorobut-2-enoate: compounds **23** and **24** could not be detected in the crude mixture, whereas two side-products of **23** were isolated. Reactions with aryl-substituted internal olefins provided variable results, whereas the reaction between 2-bromonaphthalene and a simple terminal alkene (i.e., methyl acrylate) yielded the desired product **29** with 64% yield.

To sum up, the Heck cross-coupling between aryl bromides and disubstituted alkenes is substrate-dependent. The developed reaction protocol suits different aryls and simple internal or terminal olefins. The reaction with more complex disubstituted alkenes requires further optimization, but the experimental conditions of the present work can be regarded as a favorable starting point for substrate-specific studies. The yield of the model reaction was increased from 15% (entry 12, Table 1) to 50% (Exp #14, Table 3) by tweaking different experimental conditions; therefore, it can be expected that similar results can be obtained with other substrates.

## 4 Conclusion

In the present work, we propose a green Heck reaction protocol for synthesizing trisubstituted alkenes, which are incorporated in the structure of several compounds with pharmaceutical relevance, fine chemicals and key synthetic intermediates. While the Heck reaction is one of the most popular cross-coupling reactions, and there has been extensive research to increase its efficiency and greenness, such efforts have almost always been limited to synthesizing disubstituted alkenes. Different green Heck reaction protocols are reported in the literature to access disubstituted alkenes. In this work, we screened these protocols in a model reaction to test their applicability to synthesizing the trisubstituted alkene *E*-**1**. Despite the initial limited results, a DoE study followed by further investigations helped identifying suitable conditions to guarantee the yields obtained through the “conventional” protocol, while meeting the requirements of green chemistry. In particular, the toxic and problematic solvent DMF was replaced by EtOH with equivalent yield. It is worth noting that EtOH is safer and easier to separate than DMF (i.e., by distillation during the work-up), allowing its reuse for future reactions.

The greenness of the two solvents can be easily compared by their Environmental, Health, and Safety (EHS), Life Cycle Assessment (LCA), and the net Cumulative Energy Demand (CED) (Capello et al., 2007; Byrne et al., 2016). The alcohol solvent also allows an efficient energy transfer by mw irradiation, drastically reducing reaction times compared to the original protocol (30 min vs. 5 h), proving that mw-assisted organic synthesis can be used when greening conventional synthetic approaches. However, the scalability of bench-scale mw experiments still suffers some limitations, mainly related to instrumentation. Considering the advantages of mw heating, continuous efforts are ongoing to extend its applicability on an industrial scale for organic synthesis (Dallinger et al., 2011; Egami and Hamashima, 2019; Chan et al., 2021; Martina et al., 2021). Of note is that industrial microwave heating systems have been developed, mainly in the food and materials industry, corroborating the idea that this technology can be applied to large-scale processes (Industrial Microwave Heating, 2022). Furthermore, a supported Pd catalyst allows easy recycling and reduces product contamination. Notably, even if partial leaching of the metal is still possible with the commercial catalyst used in this work, following the release and recapture mechanism typical of many solid catalysts, both the resistance to leaching and the efficiency of the redeposition are higher when using alcoholic solvents in place of coordinating solvents such as DMF (Pagliaro et al., 2012). The DoE approach has proven to be a useful tool, allowing us to draw important considerations on the effective influence of different factors on the reaction yield. For example, the composition of the solvent was crucial, while its volume and the amount of other reagents had a lower impact. Interestingly, the amount of the catalyst was irrelevant (within the experimented range) and, therefore, could be used at its lower limit. The study of the side-products evidenced that different impurities are obtained, which can be separated from the desired product *E*-**1** by column chromatography, although they are difficult to obtain as pure fractions. However, the mixture containing *Z*-**1** and **3** can be subjected to an isomerization reaction that converts them into the thermodynamically more stable trisubstituted conjugated alkene. This transformation is catalyzed by iodine, a cheap and environmentally friendly reagent with broad applicability in the industry (Yusubov and Zhdankin, 2015). Furthermore, the reaction is performed in a green solvent (*i*-PrOH) under mw irradiation, which allows the recovery of part of the waste generated during the Heck reaction. Finally, the substrate scope assessment showed that our protocol easily applies to the reaction between several aryl substrates and ethyl crotonate, while different alkene reagents gave more variable results. However, considering the experience of the model substrates, even the reactions with lower yields may represent a viable starting point for developing an efficient procedure by carefully tweaking the reaction conditions.

To conclude, in the present work, we developed a greener method for the synthesis of *E*-**1**, intermediate in the synthesis of RC-106, our in-house developed pan-sigma modulator endowed with promising anticancer activity (Tesei et al., 2019; Cortesi et al., 2020). As this hit compound is undergoing further biological investigations to assess its viability as a chemotherapeutic agent, developing an efficient, scalable, and sustainable synthesis is crucial. We believe that the newly developed synthetic protocol holds promise for the application to other structurally related products, thus broadening the applicability of green Heck reactions.

## Data Availability

The original contributions presented in the study are included in the article/Supplementary Material, further inquiries can be directed to the corresponding author.
