# Supplementary material for: A green Heck reaction protocol towards trisubstituted alkenes, versatile pharmaceutical intermediates
Source: Front Chem. 2024 Jul 10;12:1431382. doi: 10.3389/fchem.2024.1431382 (PMC11266092; doi:10.3389/fchem.2024.1431382)

Supplementary Material

# NMR characterization of *E*-1

## ^1^H-NMR


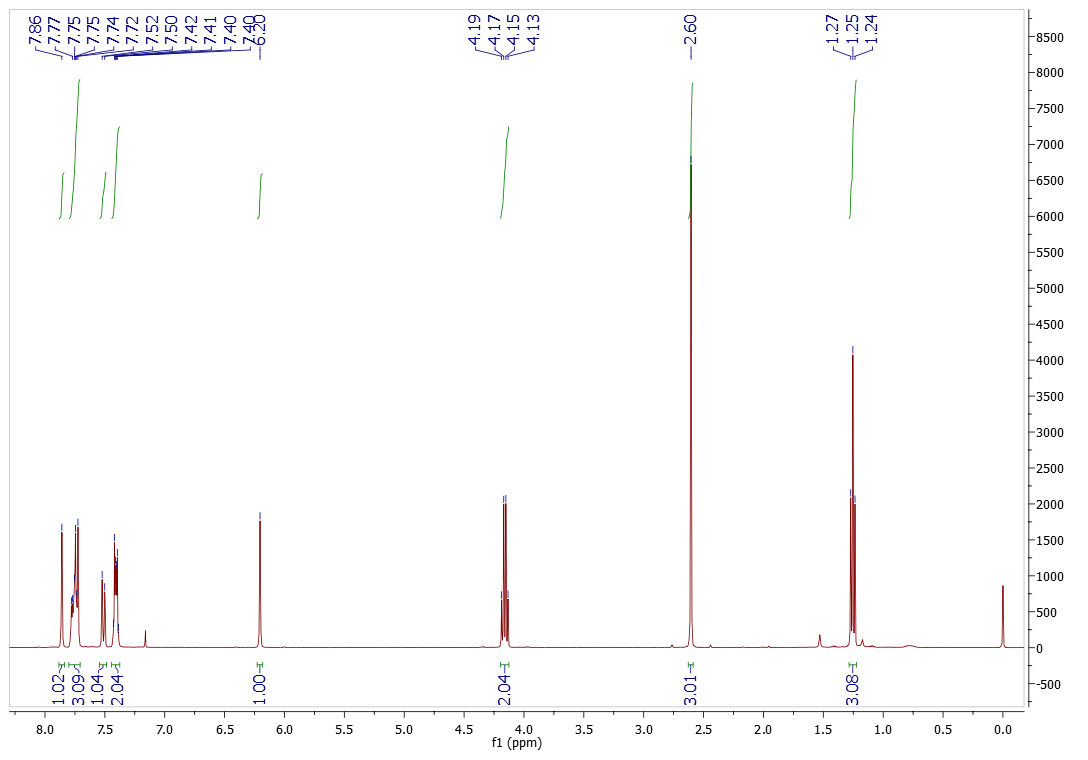


## ^13^C-NMR


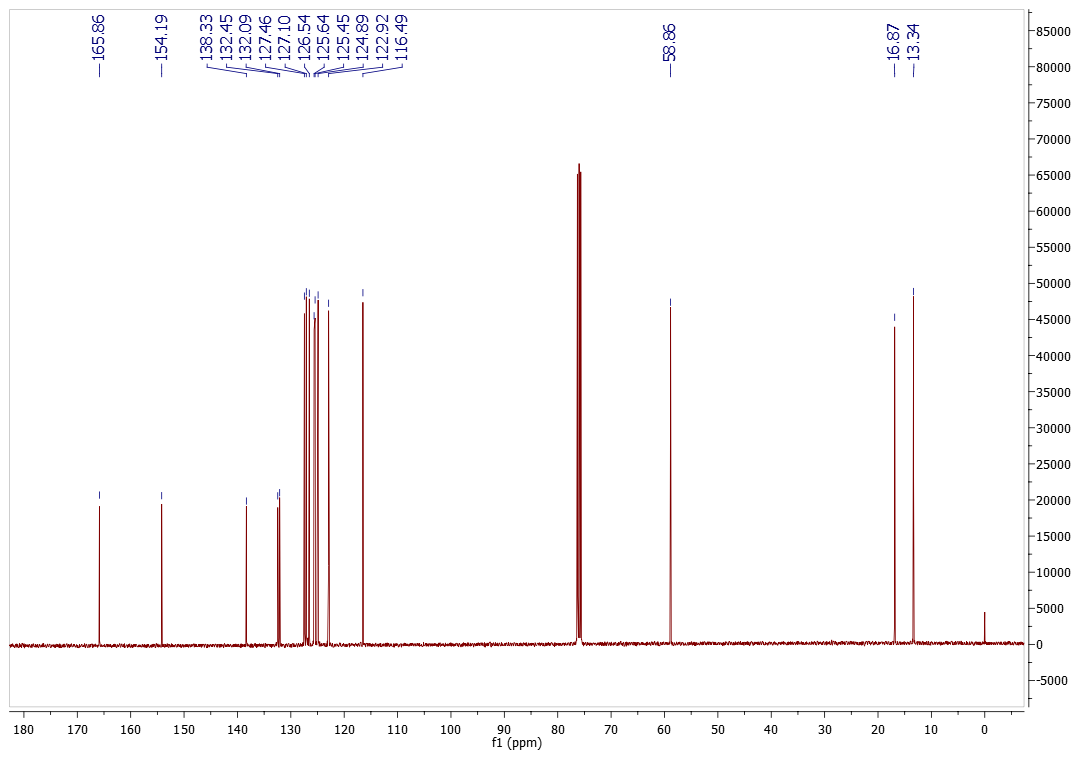


## COSY


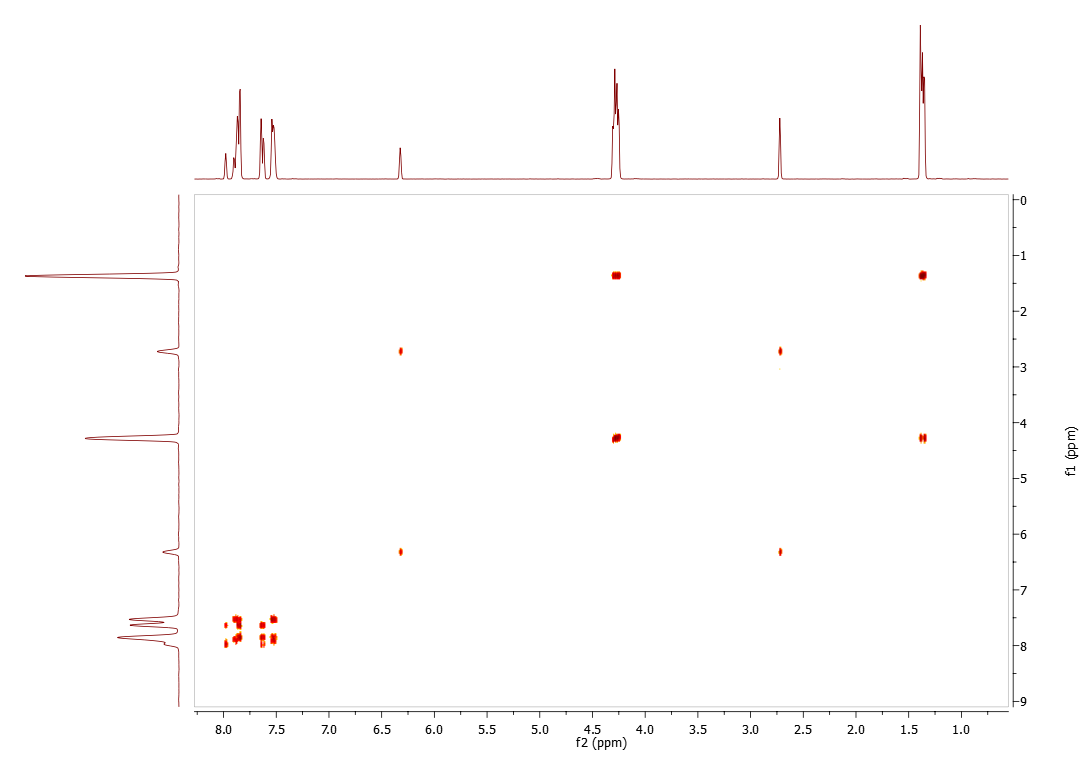


## NOESY

**
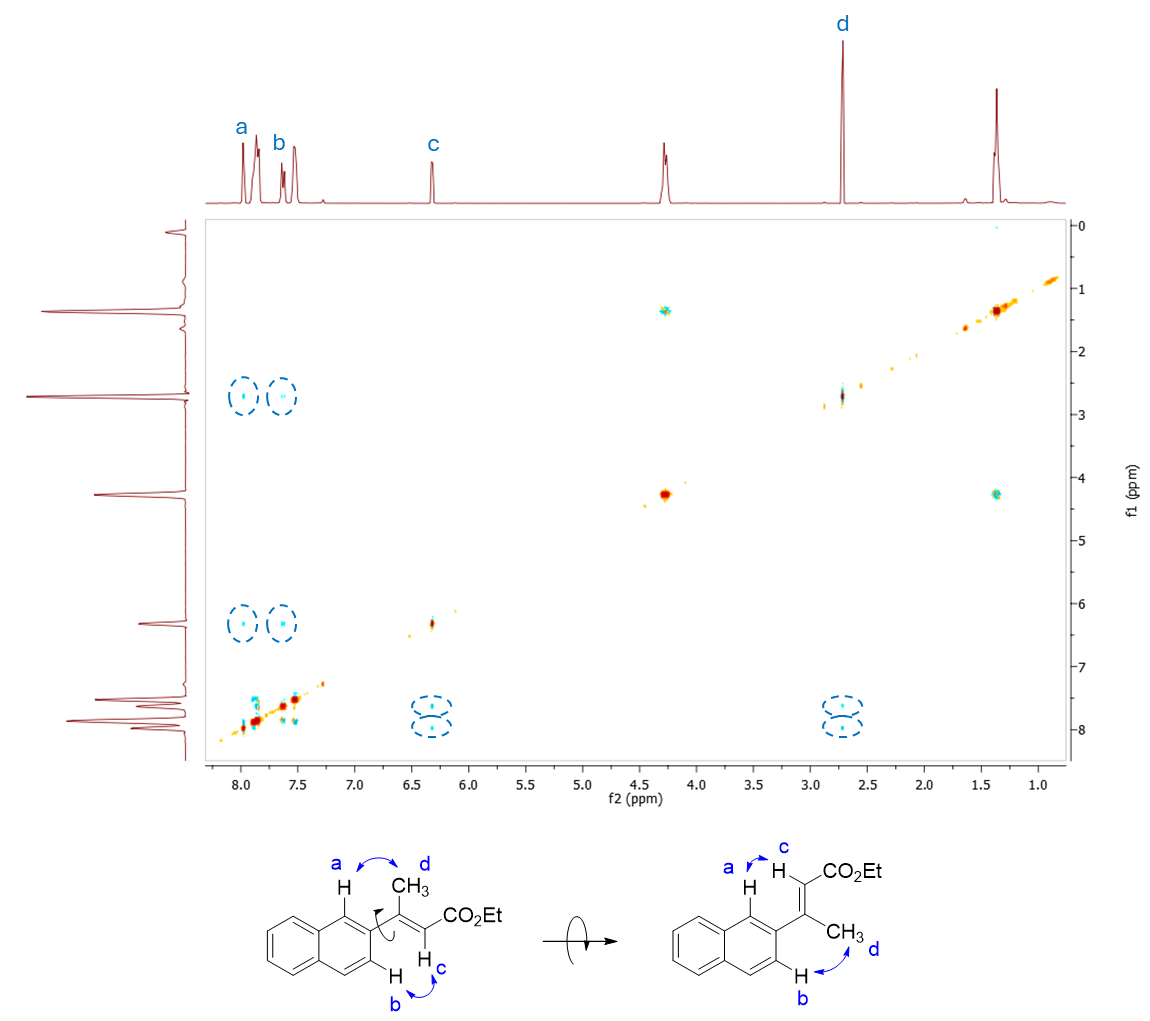
**

# DoE matrix

Herein is reported the experimental matrix obtained with the 8-factor Plackett-Burman design.

| **Exp#** | **x1** | **x2** | **x3** | **x4** | **x5** | **x6** | **e1** |
| --- | --- | --- | --- | --- | --- | --- | --- |
| *1* | 1 | 1 | 1 | -1 | 1 | -1 | -1 |
| *2* | -1 | 1 | 1 | 1 | -1 | 1 | -1 |
| *3* | -1 | -1 | 1 | 1 | 1 | -1 | 1 |
| *4* | 1 | -1 | -1 | 1 | 1 | 1 | -1 |
| *5* | -1 | 1 | -1 | -1 | 1 | 1 | 1 |
| *6* | 1 | -1 | 1 | -1 | -1 | 1 | 1 |
| *7* | 1 | 1 | -1 | 1 | -1 | -1 | 1 |
| *8* | -1 | -1 | -1 | -1 | -1 | -1 | -1 |

# DoE table

The experiments are herein listed in the order in which they were performed. The random order was used to avoid systematic errors. Two additional experiments were performed (Center1 and 2) for model validation.

|  | **x1** | **x2** | **x3** | **x4** | **x5** | **x6** |
| --- | --- | --- | --- | --- | --- | --- |
| **Exp#** | **catalyst (mg)** | **ethyl crotonate (µL)** | **Et_4_NCl (mg)** | **NaOAc (mg)** | **EtOH/H_2_O** | **solvent (mL)** |
| *6* | 20 | 60 | 250 | 50 | 1:9 | 5 |
| *2* | 10 | 100 | 250 | 100 | 1:9 | 5 |
| *4* | 20 | 60 | 50 | 100 | 9:1 | 5 |
| *8* | 10 | 60 | 50 | 50 | 1:9 | 2 |
| *3* | 10 | 60 | 250 | 100 | 9:1 | 2 |
| *Center1* | 15 | 80 | 150 | 75 | 1:1 | 3.5 |
| *1* | 20 | 100 | 250 | 50 | 9:1 | 2 |
| *7* | 20 | 100 | 50 | 100 | 1:9 | 2 |
| *Center2* | 15 | 80 | 150 | 75 | 1:1 | 3.5 |
| *5* | 10 | 100 | 50 | 50 | 9:1 | 5 |

# Isomerization reaction

The iodine-catalyzed isomerization performed on the mixture of byproducts was monitored by HPLC under the following conditions. Merck Purospher®STAR RP-18 endcapped 3 μm (100 x 2.1 mm) column; solvent A: H_2_O + 0.1% CH_2_O_2_, solvent B: ACN + 0.1% CH_2_O_2_; V_inj_: 1 μl; flow: 1 ml/min; λ= 254 nm. Gradient set as follows.

| **Time** | **A%** | **B%** |
| --- | --- | --- |
| 0.00 | 60.00 | 40.00 |
| 0.10 | 60.00 | 40.00 |
| 11.00 | 50.00 | 50.00 |
| 16.00 | 20.00 | 80.00 |
| 21.00 | 20.00 | 80.00 |
| 21.10 | 60.00 | 40.00 |
| 30.00 | 60.00 | 40.00 |

*Reaction mixture before isomerization.*


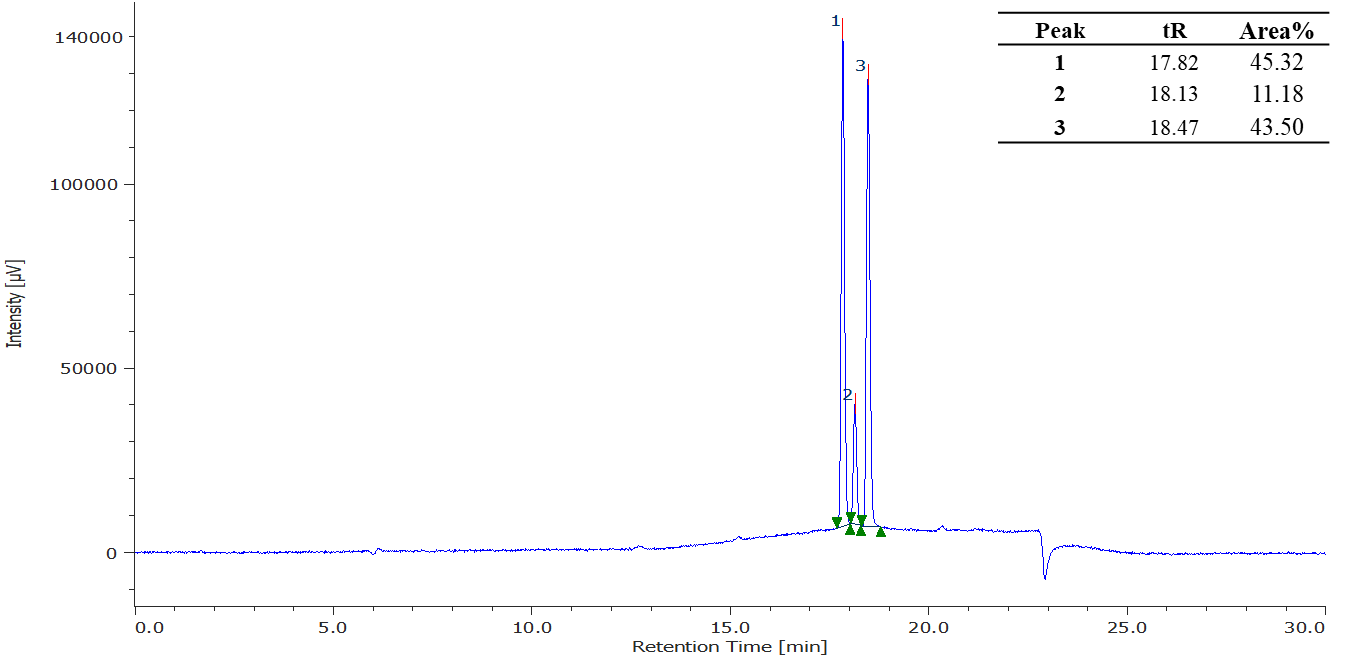


*Reaction mixture after isomerization (Table 4, Entry 7). Peak 4 corresponds to E-****1****.*

*
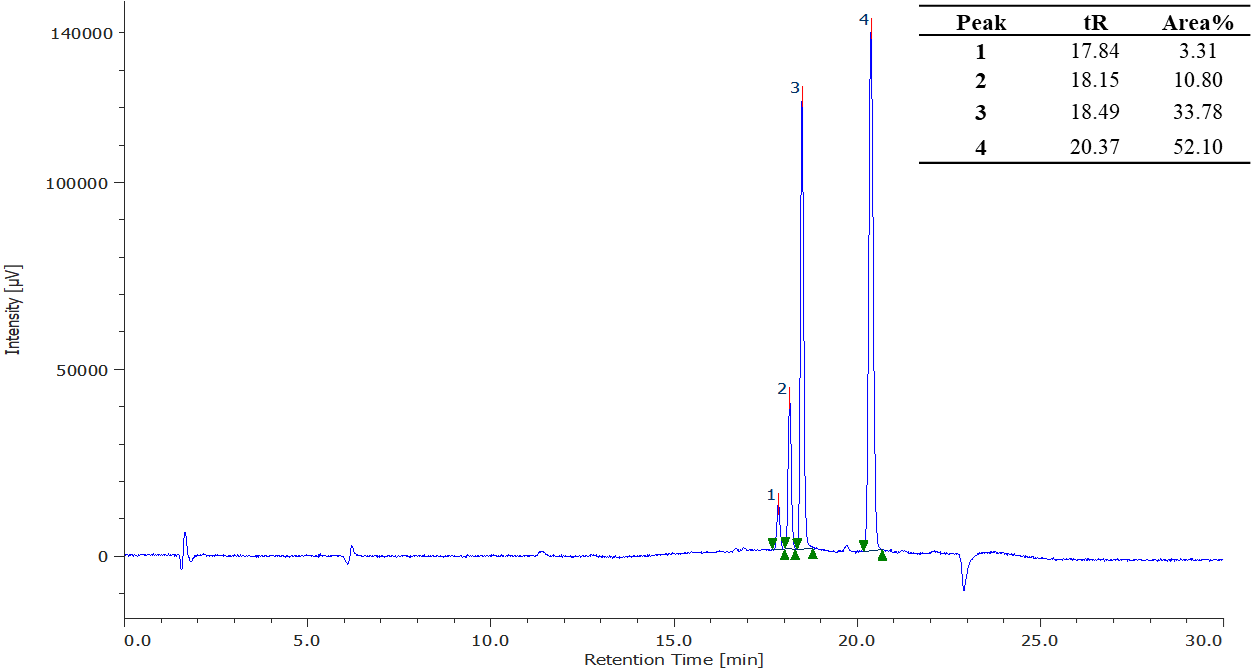
*

# Synthesis of α,β-unsaturated esters for substrate scope.

*General method for esterification of commercial acid precursors.*

The α,β-unsaturated carboxylic acid was solubilized in 5 mL of absolute EtOH, into a vial suitable for mw irradiation. A catalytic amount of concentrated H_2_SO_4_ was added, and the mixture was heated in the mw oven at 120 °C for 3 minutes, under vigorous stirring. The mixture was then diluted with Et_2_O and washed with Na_2_CO_3_ aqueous solution. The organic phase was dried over Na_2_SO_4_, filtered, and distilled at ambient pressure to remove the solvent. The product thus obtained was used for the Heck reactions without further purification.

*Ethyl (E)-pent-2-enoate*. Colorless oil. 2.5 g of product were obtained starting from 2 mL of (*E*)-pent-2-enoic acid (quantitative yield). R_f_: 0.3 (hexane/AcOEt 98:2 v/v). ^1^H NMR (400 MHz, CDCl_3_) δ 6.95 (dt, *J* = 15.7, 6.4 Hz, 1H, CH_2_C*H*=CHCO), 5.74 (dt, *J* = 15.7, 1.7 Hz, 1H, CH_2_CH=C*H*CO), 4.12 (q, *J* = 7.1 Hz, 2H, OC*H_2_*CH_3_), 2.18 – 2.14 (m, 2H, CH_3_C*H_2_*CH=CH), 1.22 (t, *J* = 7.1 Hz, 3H, OCH_2_C*H_3_*), 1.00 (t, *J* = 7.4 Hz, 3H, C*H_3_*CH_2_CH=CH).

*Ethyl (E)-4-methylpent-2-enoate*. Colorless oil. 2.0 g of product were obtained starting from 2 mL of (*E*)-4-methylpent-2-enoic acid (82% yield). R_f_: 0.6 (hexane/AcOEt 98:2 v/v). ^1^H NMR (400 MHz, CDCl_3_) δ 6.88 (dd, *J* = 15.7, 6.6 Hz, 1H, C*H*=CHCO), 5.70 (dd, *J* = 15.7, 1.4 Hz, 1H, CH=C*H*CO), 4.12 (q, *J* = 7.1 Hz, 2H, OC*H_2_*CH_3_), 2.41 – 2.38 (m, 1H, (CH_3_)_2_C*H*CH=CH), 1.22 (t, *J* = 7.1 Hz, 3H, OCH_2_C*H_3_*), 1.00 (d, *J* = 6.8 Hz, 6H, (C*H_3_*)_2_CH).

*Ethyl cinnamate*. Colorless oil. 567 mg of product were obtained starting from 500 mg of cinnamic acid (95% yield). R_f_: 0.45 (hexane/AcOEt 98:2 v/v). ^1^H NMR (400 MHz, CD_3_OD) δ 7.67 (d, *J* = 16.0 Hz, 1H, Ar–C*H*=CH), 7.60 – 7.58 (m, 2H, Ar), 7.41 – 7.37 (m, 3H, Ar), 6.51 (d, *J* = 16.0 Hz, 1H, CH=C*H*CO_2_Et), 4.23 (q, *J* = 7.1 Hz, 2H, OC*H_2_*CH_3_), 1.31 (t, *J* = 7.1 Hz, 3H, OCH_2_C*H_3_*).

*Ethyl (E)-3-(4-nitrophenyl)acrylate*. Yellow solid. 506 mg of product were obtained starting from 507 mg of (*E*)-3-(4-nitrophenyl)acrylic acid (87% yield). R_f_: 0.4 (hexane/AcOEt 94:6 v/v). ^1^H NMR (400 MHz, CD_3_OD) δ 8.26 (d, *J* = 8.8 Hz, 2H, Ar), 7.85 (d, *J* = 8.7 Hz, 2H, Ar), 7.75 (d, *J* = 16.1 Hz, 1H, Ar–C*H*=CH), 6.71 (d, *J* = 16.1 Hz, 1H, C=C*H*CO_2_Et), 4.26 (q, *J* = 7.1 Hz, 2H, C*H_2_*CH_3_), 1.33 (t, *J* = 7.1 Hz, 3H, CH_2_C*H_3_*).

# Chromatographic methods for crudes HPLC analyses.

The following HPLC methods were used for the substrate scope study. Solvent A: H_2_O + 0.1% CH_2_O_2_, solvent B: ACN + 0.1% CH_2_O_2_); V_inj_: 1 μl; flow: 1 ml/min; λ= ideally selected for each chromatogram.

**Table S1**. Method 2 for HPLC gradient. Used for compounds **6**, **7**, **10**, **17**.

| **Time** | **A%** | **B%** |
| --- | --- | --- |
| 0.00 | 60.00 | 40.00 |
| 0.10 | 60.00 | 40.00 |
| 11.00 | 50.00 | 50.00 |
| 16.00 | 20.00 | 80.00 |
| 21.00 | 20.00 | 80.00 |
| 21.10 | 60.00 | 40.00 |
| 30.00 | 60.00 | 40.00 |

**Table S2**. Method 3 for HPLC gradient. Used for compounds **9**, **11**, **15**, **16**.

| **Time** | **A%** | **B%** |
| --- | --- | --- |
| 0.00 | 60.00 | 40.00 |
| 0.10 | 60.00 | 40.00 |
| 5.00 | 50.00 | 50.00 |
| 10.00 | 20.00 | 80.00 |
| 21.00 | 20.00 | 80.00 |
| 21.10 | 60.00 | 40.00 |
| 30.00 | 60.00 | 40.00 |

**Table S3**. Method 4 for HPLC gradient. Used for compounds **8**, **14**, **19**.

| **Time** | **A%** | **B%** |
| --- | --- | --- |
| 0.00 | 90.00 | 10.00 |
| 0.10 | 90.00 | 10.00 |
| 5.00 | 60.00 | 40.00 |
| 16.00 | 50.00 | 50.00 |
| 19.00 | 20.00 | 80.00 |
| 21.00 | 20.00 | 80.00 |
| 21.10 | 90.00 | 10.00 |
| 30.00 | 90.00 | 10.00 |

**Table S4**. Method 5 for HPLC gradient. Used for compound **13**.

| **Time** | **A%** | **B%** |
| --- | --- | --- |
| 0.00 | 60.00 | 40.00 |
| 0.10 | 60.00 | 40.00 |
| 5.00 | 40.00 | 60.00 |
| 21.00 | 20.00 | 80.00 |
| 21.10 | 60.00 | 40.00 |
| 30.00 | 60.00 | 40.00 |

**Table S5.** Method 6 for HPLC gradient. Used for compound **18**, **21**, **28**.

| **Time** | **A%** | **B%** |
| --- | --- | --- |
| 0.00 | 60.00 | 40.00 |
| 0.10 | 60.00 | 40.00 |
| 13.00 | 20.00 | 80.00 |
| 28.00 | 20.00 | 80.00 |
| 28.10 | 10.00 | 90.00 |
| 33.00 | 10.00 | 90.00 |
| 33.10 | 60.00 | 40.00 |
| 43.00 | 60.00 | 40.00 |

# ^1^H-NMR characterization of known compounds

*Ethyl (E)-3-phenylbut-2-enoate (****6****).* ^1^H-NMR (400 MHz, DMSO *d_6_*): δ 7.59 – 7.55 (m, 2H, Ar), 7.42 – 7.39 (m, 3H, Ar), 6.15 (q, *J* = 1.2 Hz, 1H, CH_3_C=C*H*), 4.15 (q, *J* = 7.1 Hz, 2H, OC*H_2_*CH_3_), 2.52 (d, *J* = 1.2 Hz, 3H, C*H_3_*C=CH ), 1.24 (t, *J* = 7.1 Hz, 3H, OCH_2_C*H_3_*).

*Ethyl (E)-3-(4-methoxyphenyl)but-2-enoate (****7****).* ^1^H-NMR (400 MHz, DMSO *d_6_*): δ 7.59 – 7.54 (m, 2H, Ar), 6.99 – 6.94 (m, 2H, Ar), 6.12 (brs, 1H, CH_3_C=C*H*), 4.14 (q, *J* = 7.1 Hz, 2H, OC*H_2_*CH_3_), 3.79 (s, 3H, OC*H_3_*), 2.51 (d, *J* = 1.2 Hz, 3H, C*H_3_*C=CH), 1.25 (t, *J* = 7.1 Hz, 3H, OCH_2_C*H_3_*).

*Ethyl (E)-3-(4-hydroxyphenyl)but-2-enoate (****8****).* ^1^H-NMR (400 MHz, DMSO *d_6_*): δ 9.81 (s, 1H, O*H*), 7.48 – 7.43 (m, 2H, Ar), 6.82 – 6.77 (m, 2H, Ar), 6.08 (brs, 1H, CH_3_C=C*H*), 4.12 (q, *J* = 7.1 Hz, 2H, OC*H_2_*CH_3_), 2.48 (d, *J* = 1.2 Hz, 3H, C*H_3_*C=CH), 1.23 (t, *J* = 7.1 Hz, 3H, OCH_2_C*H_3_*).

*Ethyl (E)-3-(4-(trifluoromethyl)phenyl)but-2-enoate (****9****).* ^1^H-NMR (400 MHz, DMSO *d_6_*): δ 7.80 – 7.76 (m, 4H, Ar), 6.23 (d, J = 1.3 Hz, 1H, CH_3_C=C*H*), 4.17 (q, *J* = 7.1 Hz, 2H, OC*H_2_*CH_3_), 2.53 (d, *J* = 1.3 Hz, 3H, C*H_3_*C=CH), 1.25 (t, *J* = 7.1, 3H, OCH_2_C*H_3_*).

*Ethyl (E)-3-(4-nitrophenyl)but-2-enoate (****10****).* ^1^H-NMR (400 MHz, DMSO *d_6_*): δ 8.26 – 8.22 (m, 2H, Ar), 7.88 – 7.83 (m, 2H, Ar), 6.29 (q, *J* = 1.3 Hz, 1H, CH_3_C=C*H*), 4.17 (q, *J* = 7.1 Hz, 2H, OC*H_2_*CH_3_), 2.53 (d, *J* = 1.3 Hz, 3H, C*H_3_*C=CH), 1.26 (t, *J* = 7.1, 3H, OCH_2_C*H_3_*).

*Ethyl (E)-3-([1,1'-biphenyl]-4-yl)but-2-enoate (****11****).* ^1^H NMR (400 MHz, CDCl_3_) δ 7.55 – 7.49 (m, 6H, Ar), 7.42 – 7.35 (m, 2H, Ar), 7.32 – 7.26 (m, 1H, Ar), 6.14 (q, *J* = 1.2 Hz, 1H, CH_3_C=C*H*), 4.16 (q, *J* = 7.1 Hz, 2H, OC*H_2_*CH_3_), 2.55 (d, *J* = 1.2 Hz, 3H, C*H_3_*C=CH), 1.26 (t, *J* = 7.1 Hz, 3H, OCH_2_C*H_3_*).

*Ethyl (E)-3-(quinolin-3-yl)but-2-enoate (****14****).* ^1^H-NMR (400 MHz, DMSO *d_6_*): δ 9.13 (d, *J* = 2.3 Hz, 1H, Ar), 8.60 (d, *J* = 2.3 Hz, 1H, Ar), 8.04 (m, 2H, Ar), 7.82 – 7.77 (m, 1H, Ar), 7.68 – 7.63 (m, 1H, Ar), 6.45 (brs, 1H, CH_3_C=C*H*), 4.19 (q, *J* = 7.1 Hz, 2H, OC*H_2_*CH_3_), 2.65 (d, *J* = 1.3 Hz, 3H, C*H_3_*C=CH), 1.27 (t, *J* = 7.1 Hz, 3H, OCH_2_C*H_3_*).

*Ethyl (E)-3-phenylpent-2-enoate (****19****).* ^1^H NMR (400 MHz, MeOD) δ 7.39 – 7.34 (m, 2H, Ar), 7.32 – 7.26 (m, 3H, Ar), 5.90 (s, 1H, CH_2_C=C*H*), 4.09 (q, *J* = 7.1 Hz, 2H, OC*H_2_*CH_3_), 3.02 (q, *J* = 7.5 Hz, 2H, CH_3_C*H_2_*C=C), 1.20 (t, *J* = 7.1 Hz, 3H, OCH_2_C*H_3_*), 0.93 (t, *J* = 7.5 Hz, 3H, C*H_3_*CH_2_C=C).

*Ethyl (E)-4-methyl-3-phenylpent-2-enoate (****21****).* ^1^H NMR (400 MHz, CDCl_3_) δ 7.35 – 7.33 (m, 3H, Ar), 7.23 – 7.21 (m, 2H, Ar), 5.72 (s, 1H, CHC=C*H*), 4.22 (q, *J* = 7.1 Hz, 2H, OC*H_2_*CH_3_), 4.13 (m, 1H, (CH_3_)_2_C*H*), 1.33 (t, *J* = 7.1 Hz, 3H, OCH_2_C*H_3_*), 1.11 (d, *J* = 7.0 Hz, 6H, (C*H_3_*)_2_CH).

*Ethyl (E)-3-(naphthalen-2-yl)-3-phenylacrylate (****25****).* ^1^H NMR (400 MHz, CDCl_3_) δ 7.76 – 7.68 (m, 3H, Ar), 7.61 (brs, 1H, Ar), 7.45 – 7.39 (m, 3H, Ar), 7.36 – 7.34 (m, 3H, Ar), 7.21 – 7.20 (m, 2H, Ar), 6.43 (s, 1H, C=C*H*), 4.00 (q, *J* = 7.1 Hz, 2H, OC*H_2_*CH_3_), 1.06 (t, *J* = 6.9 Hz, 3H, OCH_2_C*H_3_*).

*Ethyl (E)-3-(4-nitrophenyl)-3-phenylacrylate (****26****).* ^1^H NMR (400 MHz, CDCl_3_) δ 8.20 (d, *J* = 8.8 Hz, 2H, Ar), 7.34 – 7.27 (m, 5H, Ar), 7.19 – 7.17 (m, 2H, Ar), 6.41 (s, 1H, C=C*H*), 4.00 (q, *J* = 7.1 Hz, 4H, OC*H_2_*CH_3_), 1.09 (t, *J* = 7.1 Hz, 3H, OCH_2_C*H_3_*).

*Ethyl 3,3-diphenylacrylate (****27****).* ^1^H NMR (400 MHz, CDCl_3_) δ 7.33 – 7.21 (m, 8H, Ar), 7.16 – 7.14 (m, 2H, Ar), 6.29 (s, 1H, C=C*H*), 3.98 (q, *J* = 7.1 Hz, 2H, OC*H_2_*CH_3_), 1.04 (t, *J* = 7.1 Hz, 3H, OCH_2_C*H_3_*).

*Methyl (E)-3-(naphthalen-2-yl)acrylate (****29****).* ^1^H NMR (400 MHz, CDCl_3_) δ 7.87 (s, 1H, Ar), 7.82 – 7.75 (m, 4H, Ar), 7.61 (dd, *J* = 8.6, 1.5 Hz, 1H), 7.48 – 7.42 (m, 2H), 6.49 (d, *J* = 16.0 Hz, 1H, C=C*H*), 3.77 (s, 3H, OC*H_3_*).

# ^1^H-NMR and ^13^C-NMR spectra of new compounds.

*Ethyl (E)-3-(6-methoxynaphthalen-2-yl)but-2-enoate (****12****)*.


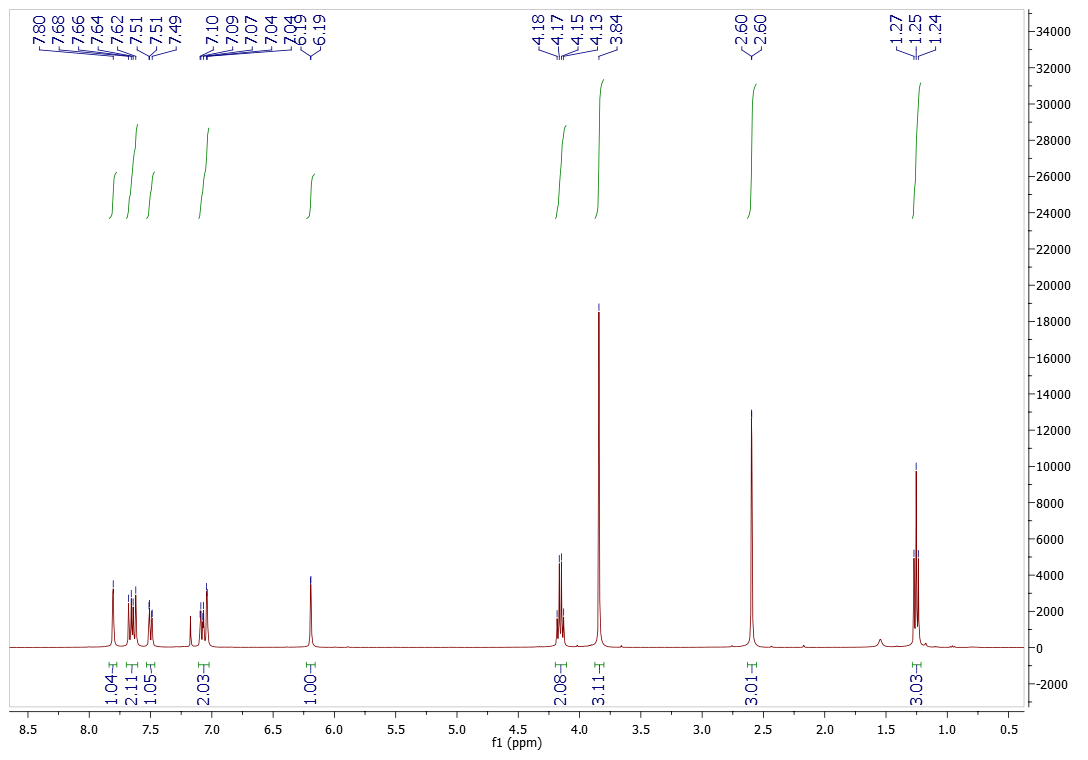


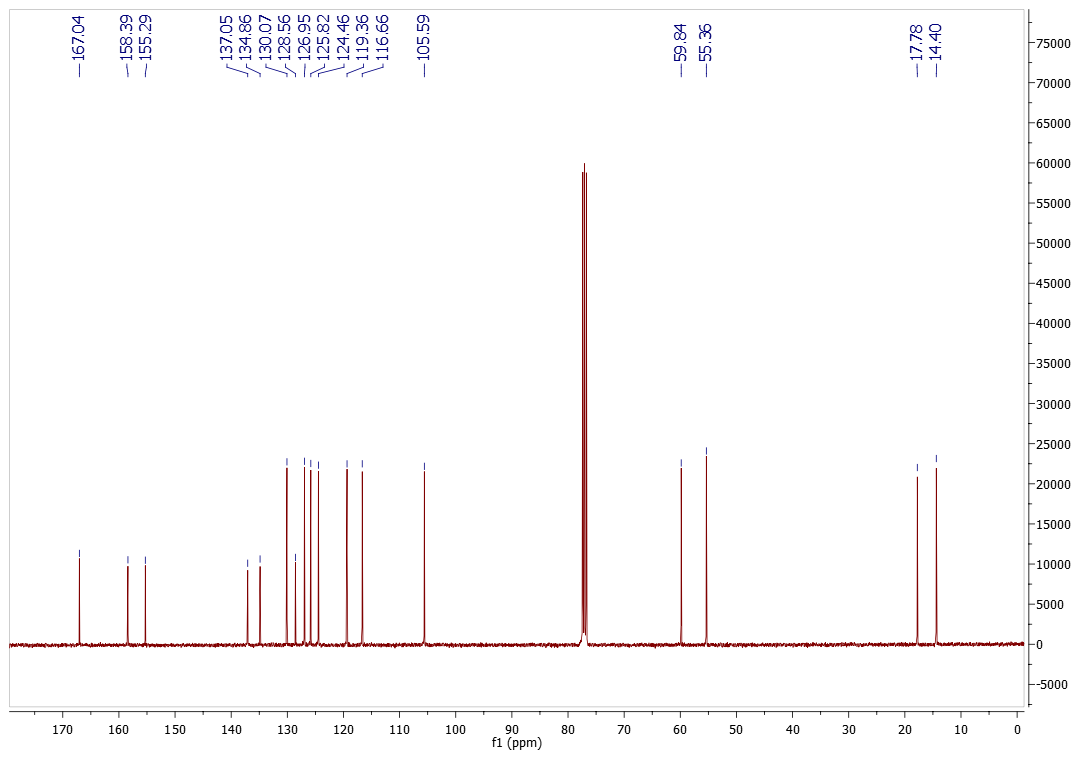


*Ethyl (E)-3-(6-hydroxynaphthalen-2-yl)but-2-enoate (****13****).*


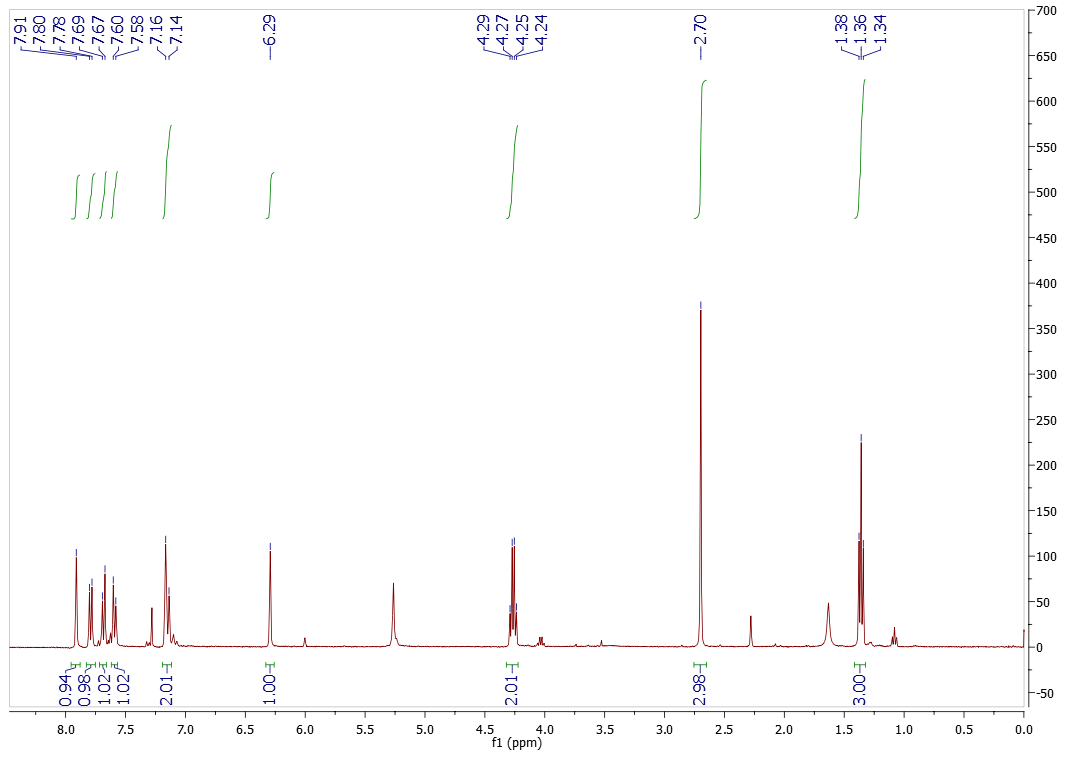


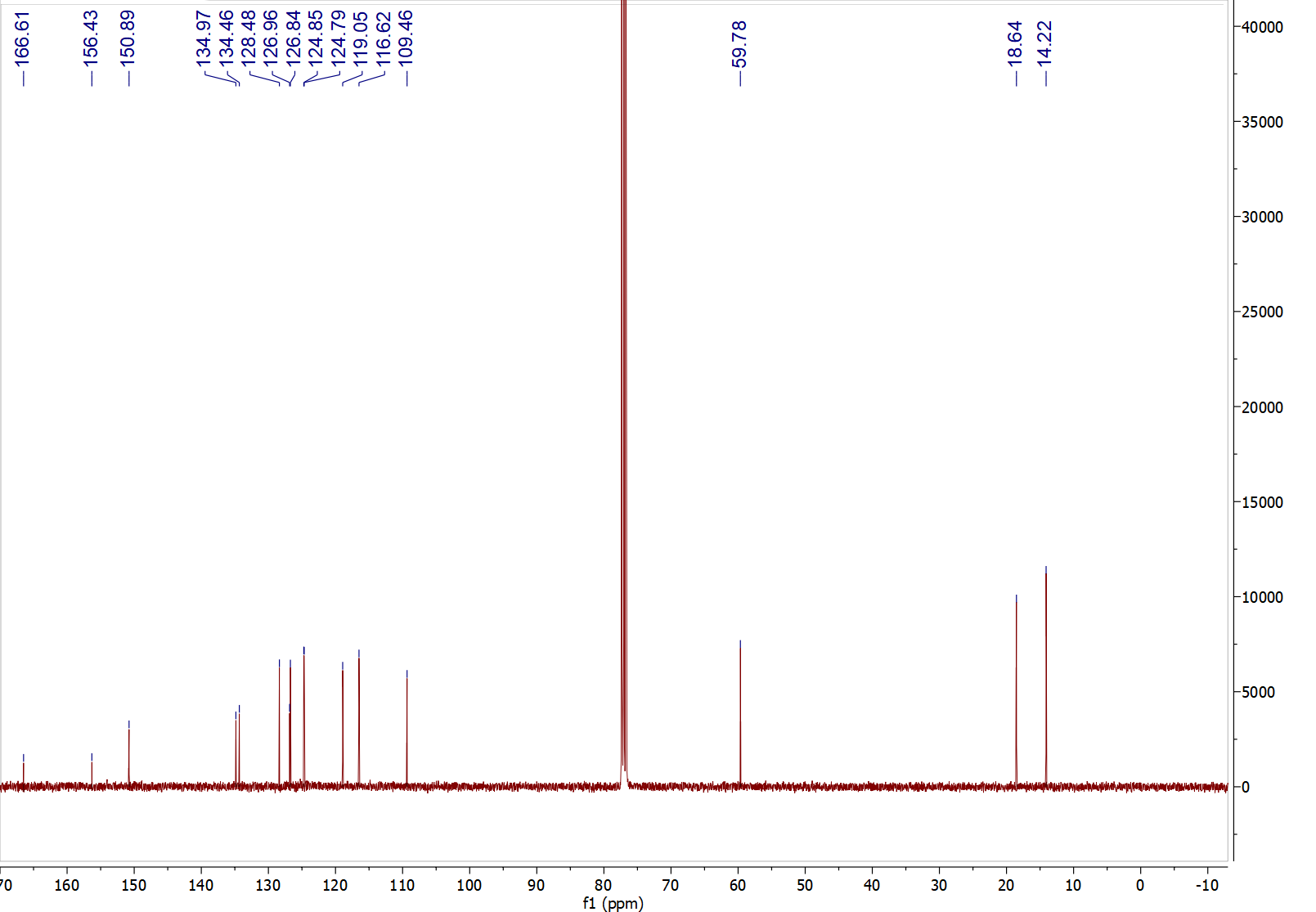


*Ethyl (E)-3-(6-(benzyloxy)naphthalen-2-yl)but-2-enoate (****15****).*


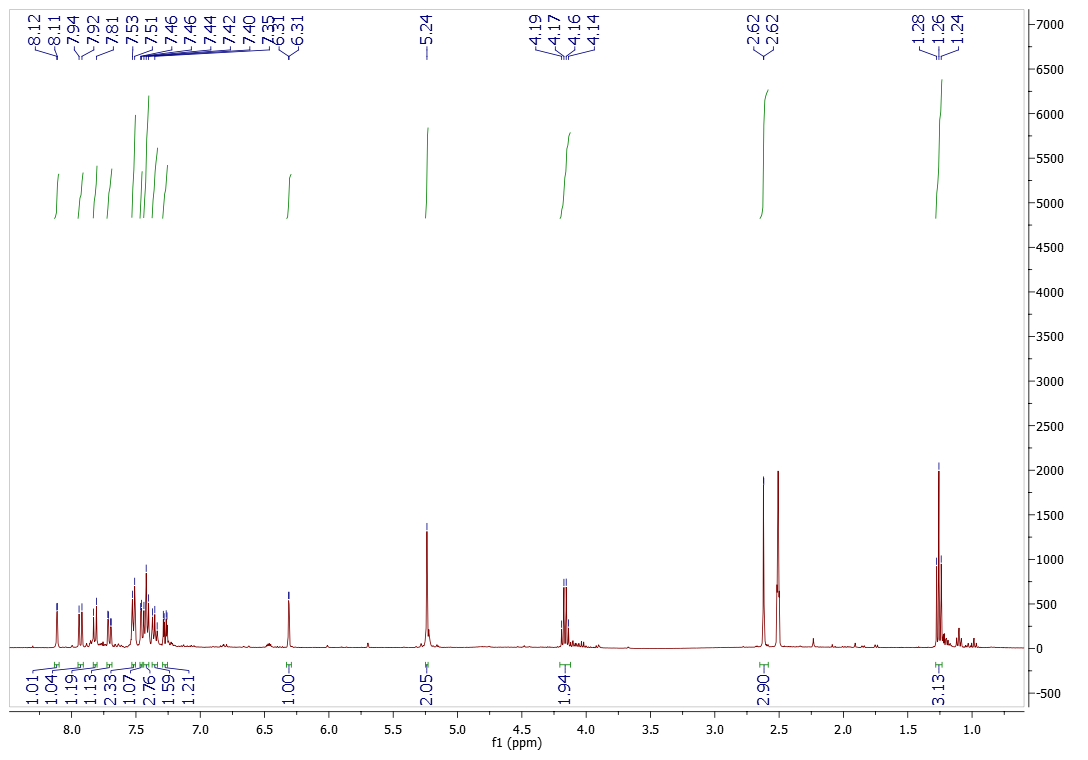


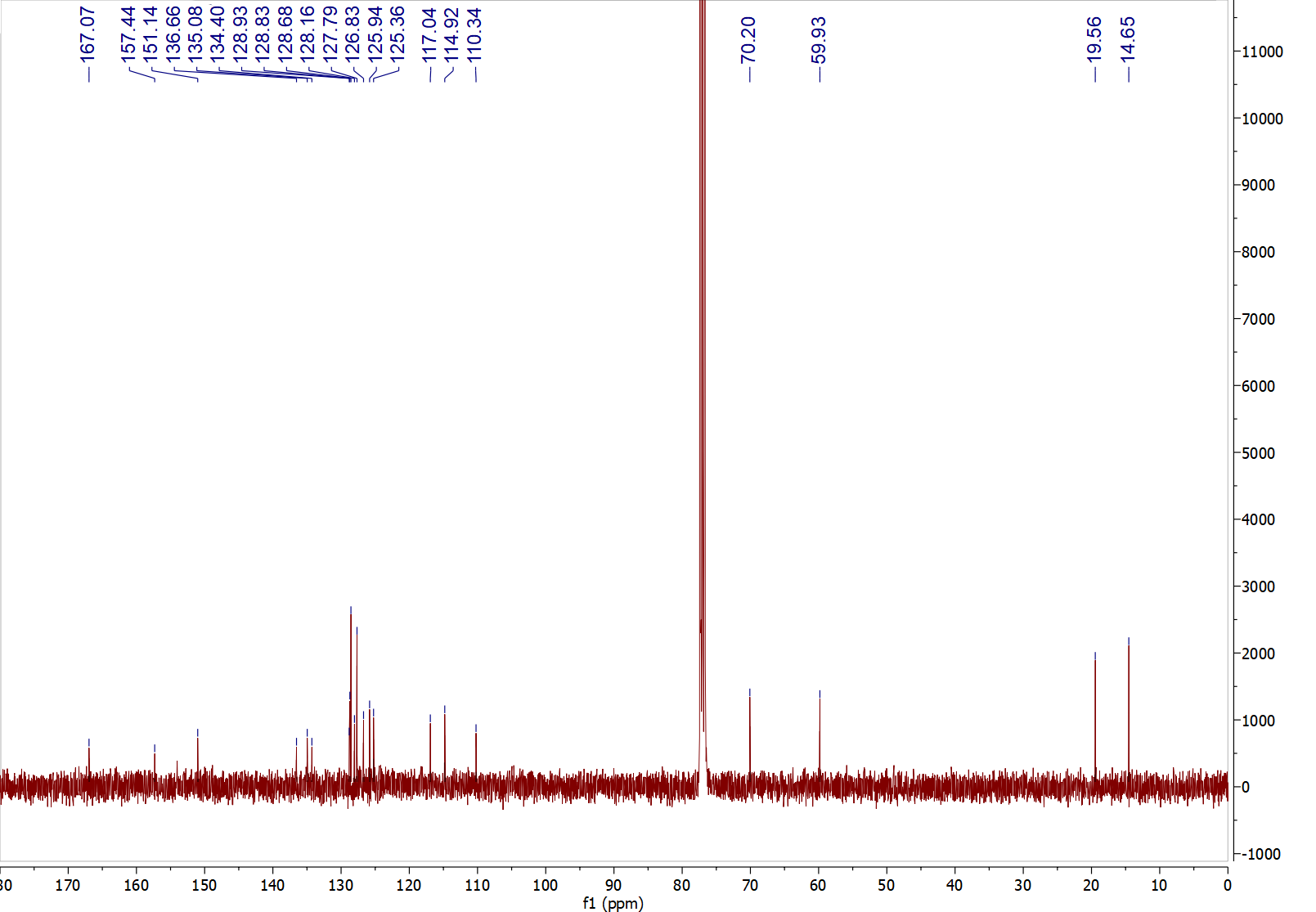


*Ethyl (E)-3-(phenanthren-9-yl)but-2-enoate (****16****).*


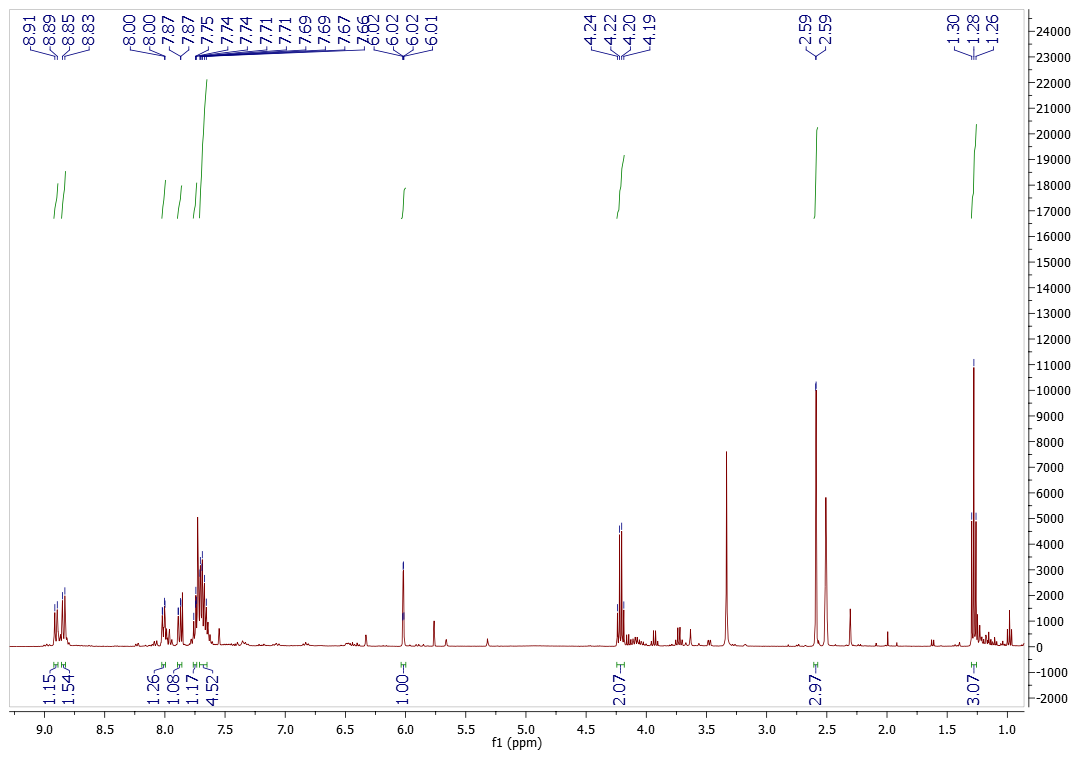


*
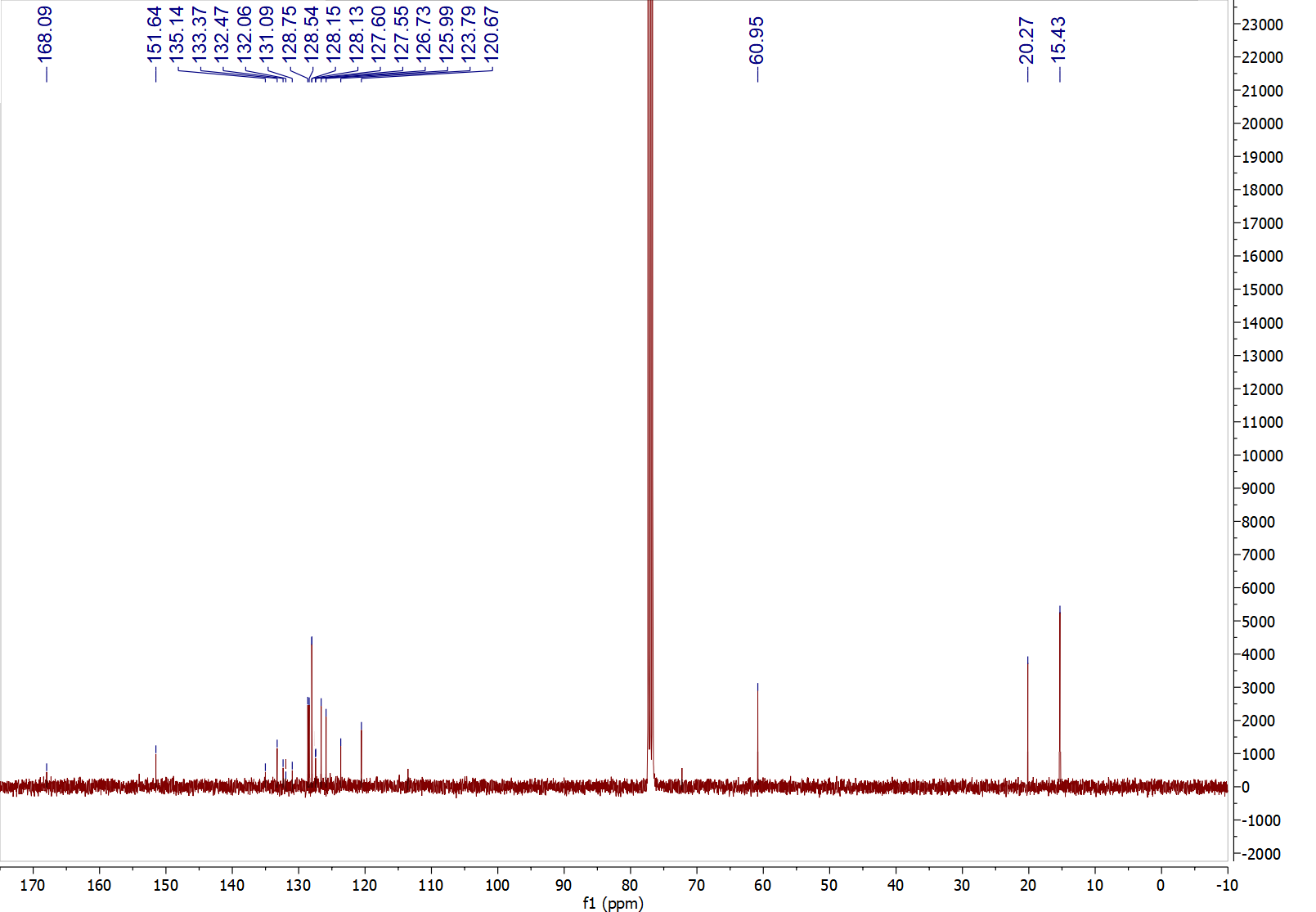
*

*Ethyl (E)-3-(5-acetylthiophen-2-yl)but-2-enoate (****17****).*


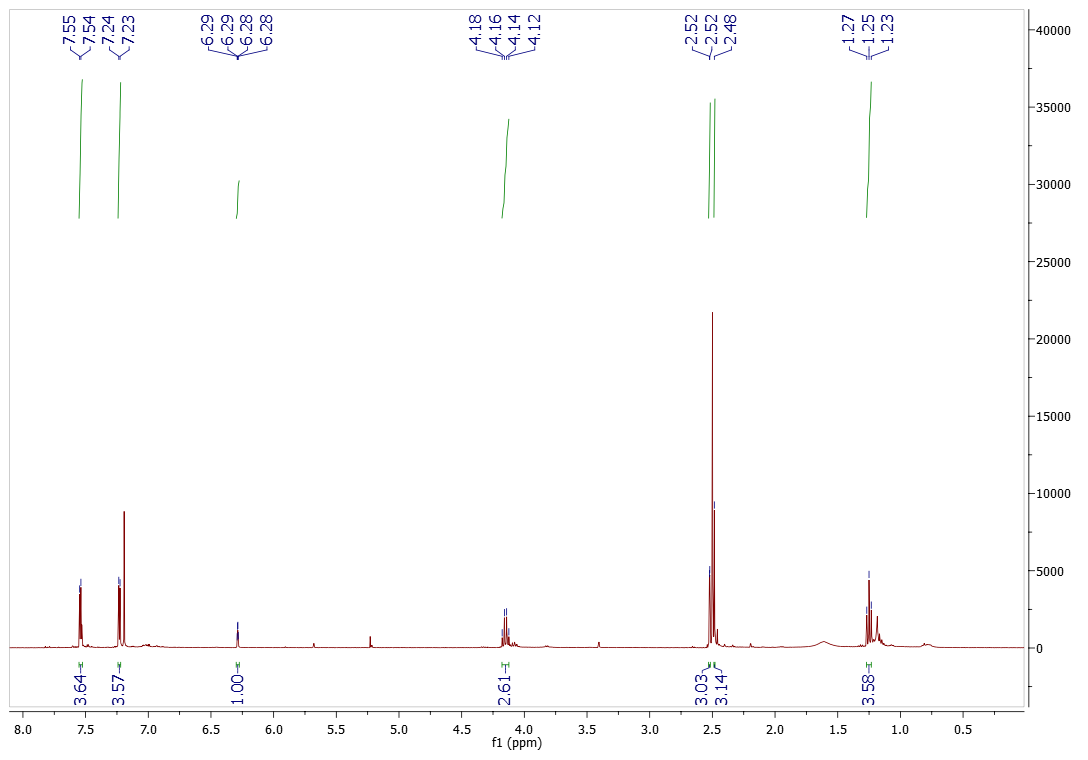


*
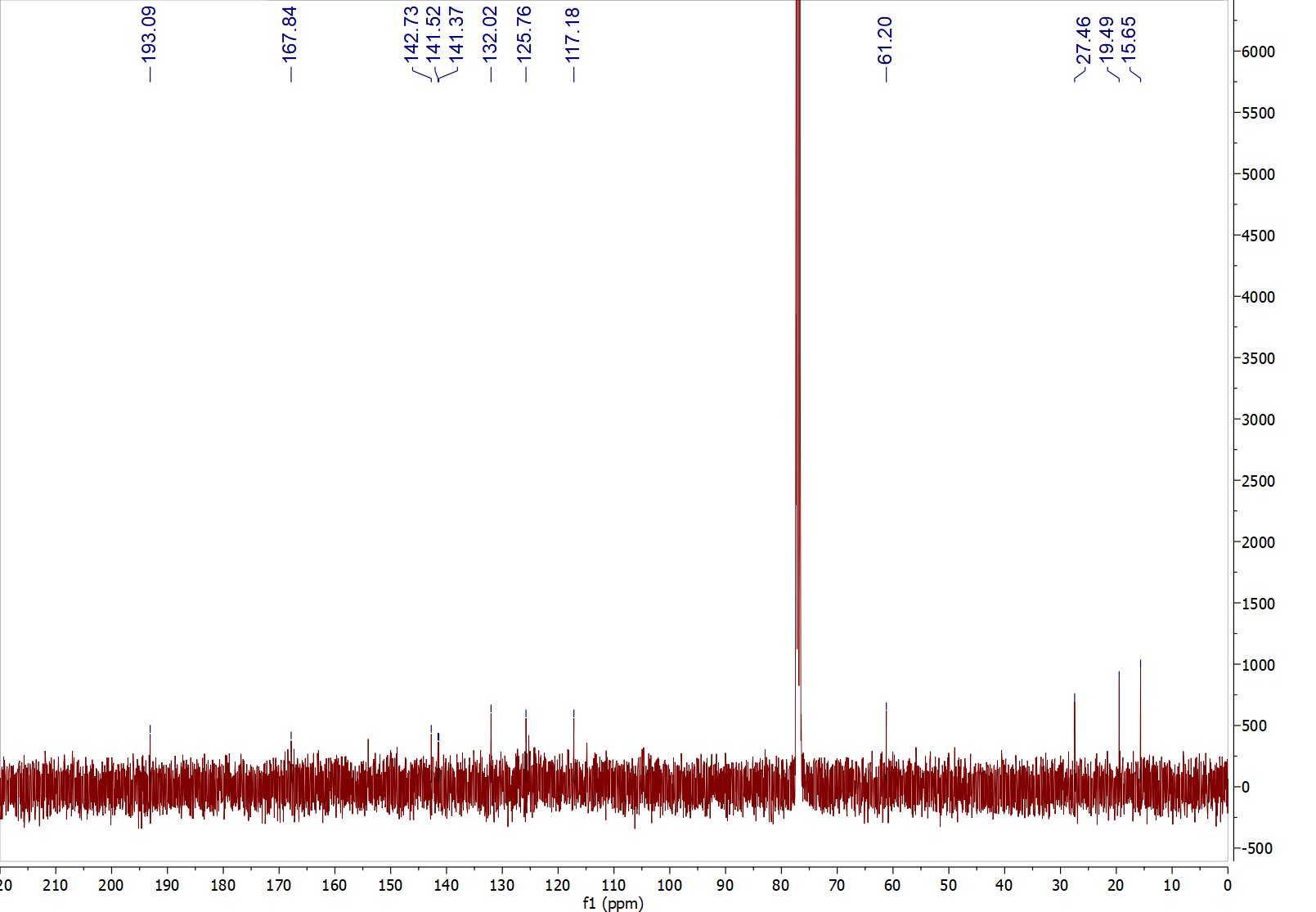
*

*Ethyl (E)-3-(naphthalen-2-yl)pent-2-enoate (****18****).*


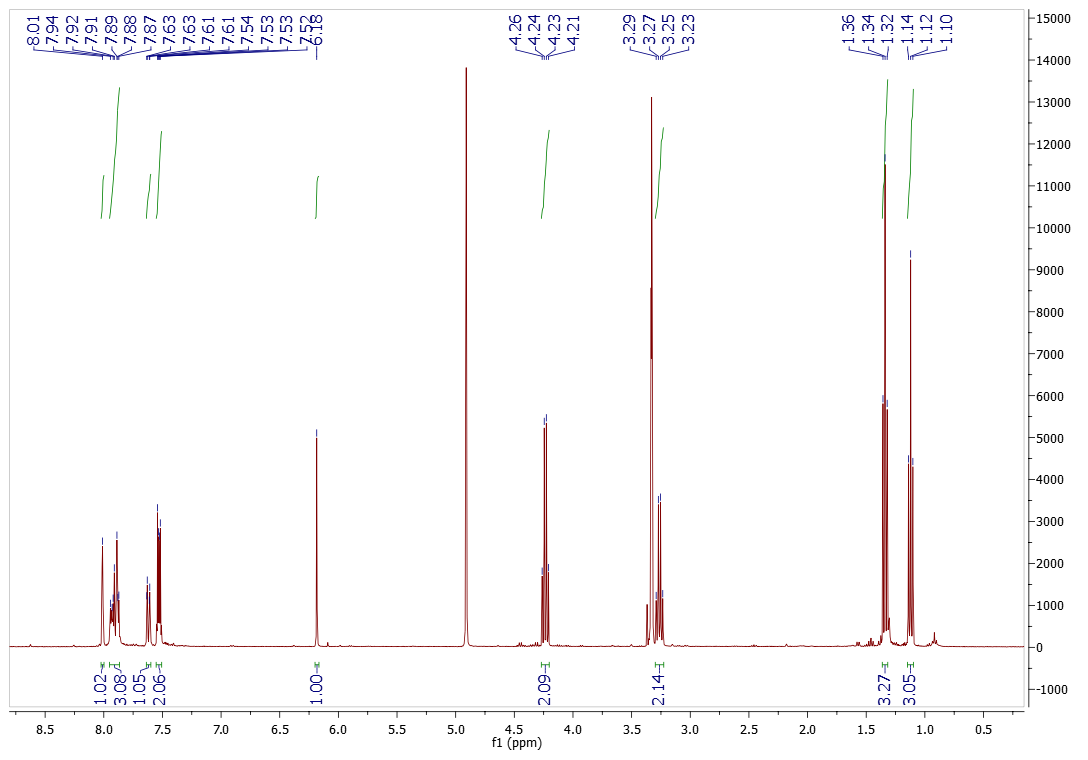


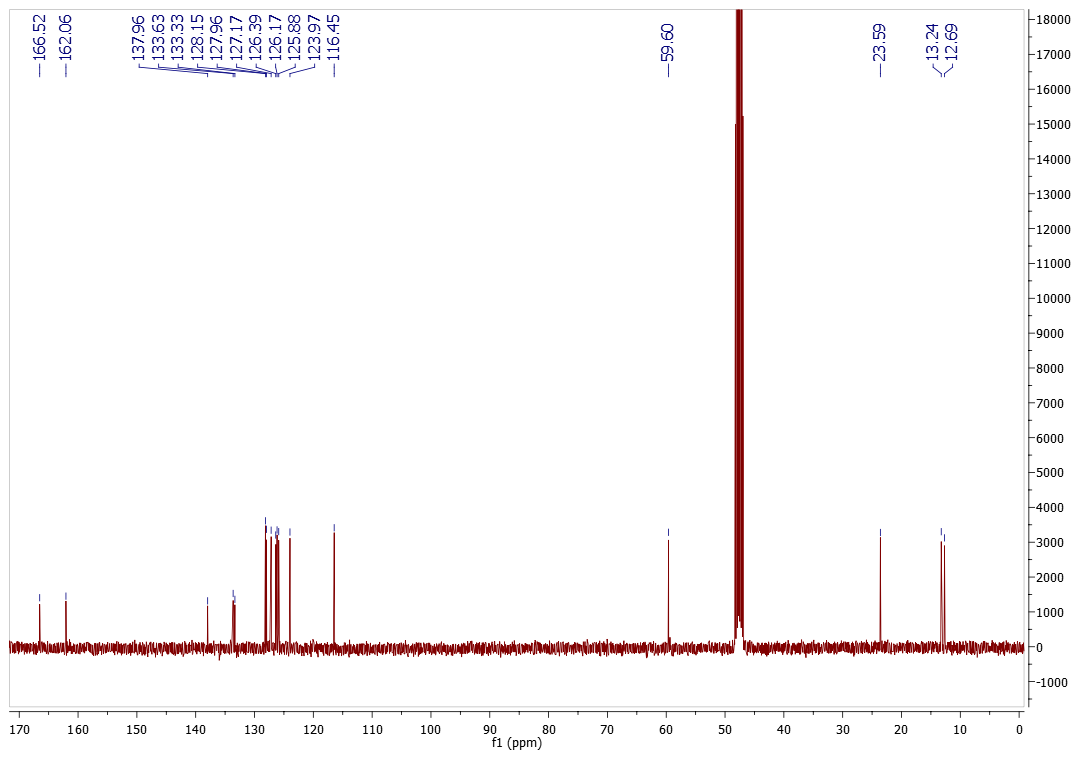


*Ethyl (E)-4-methyl-3-(naphthalen-2-yl)pent-2-enoate (****20****).*


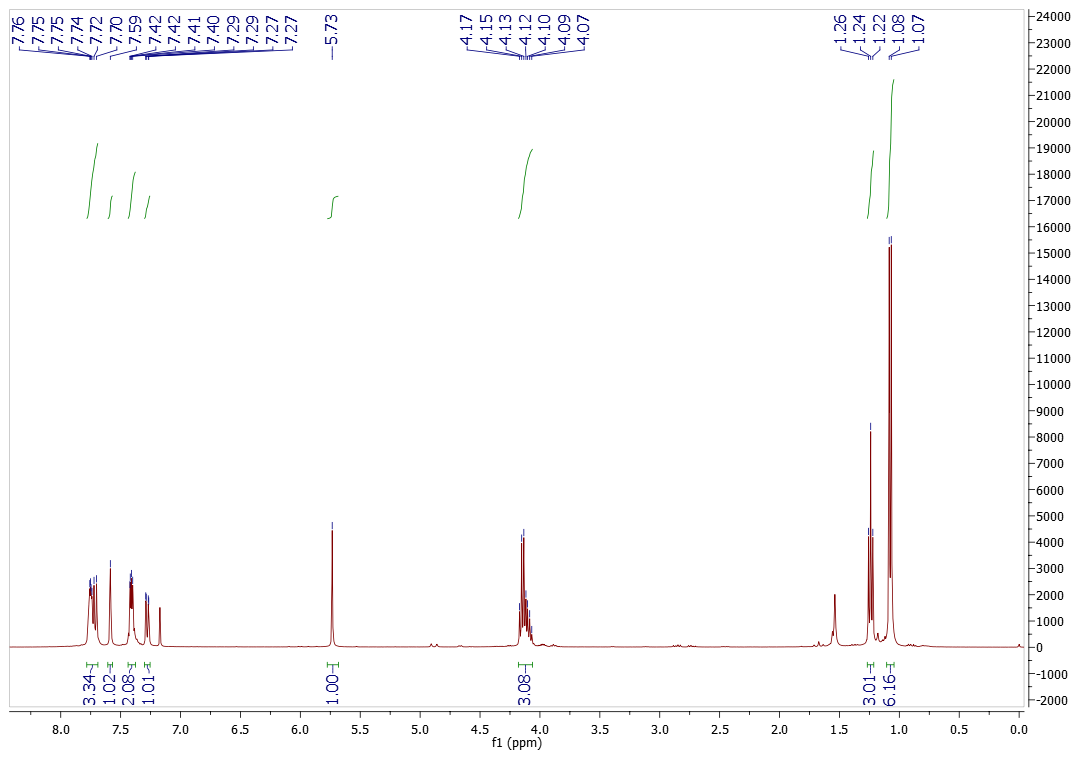


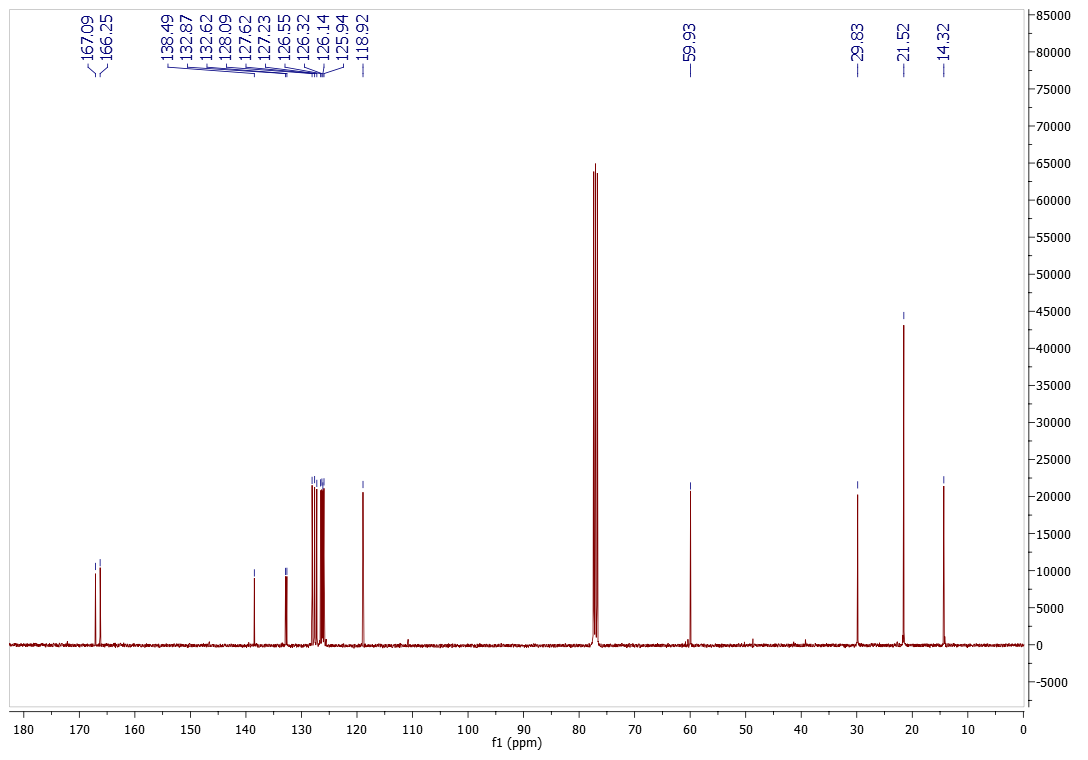


*Ethyl (Z)-4,4,4-trifluoro-3-(naphthalen-2-yl)but-2-enoate (****22****).*


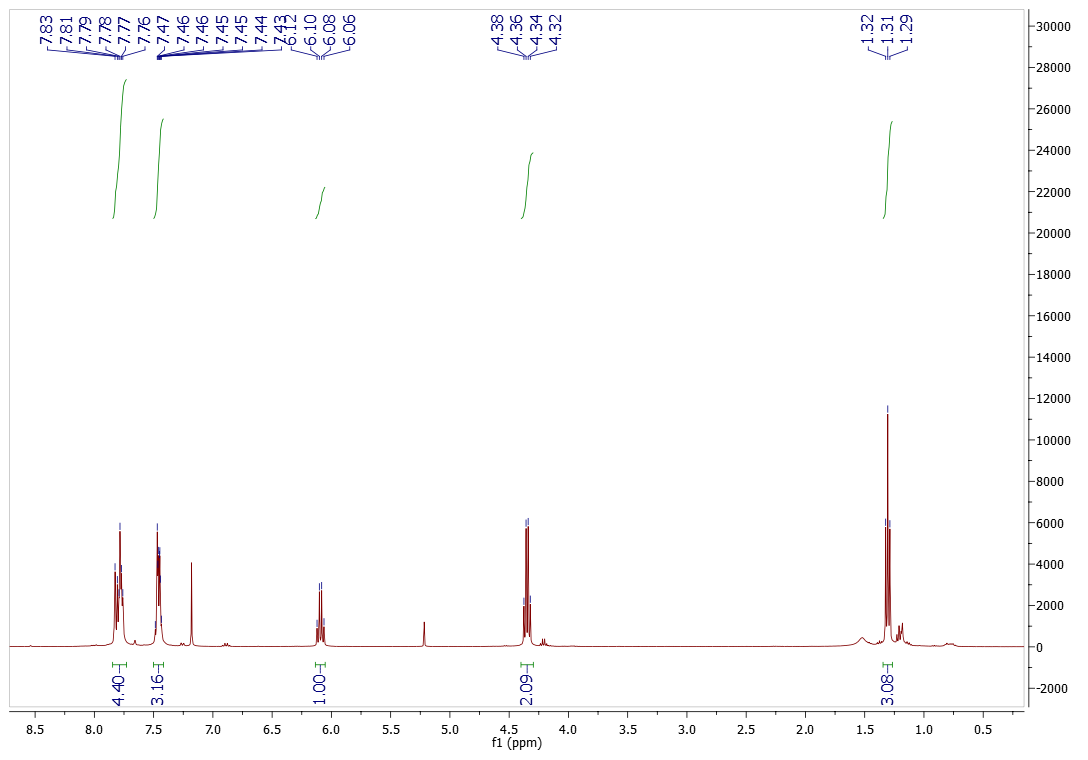


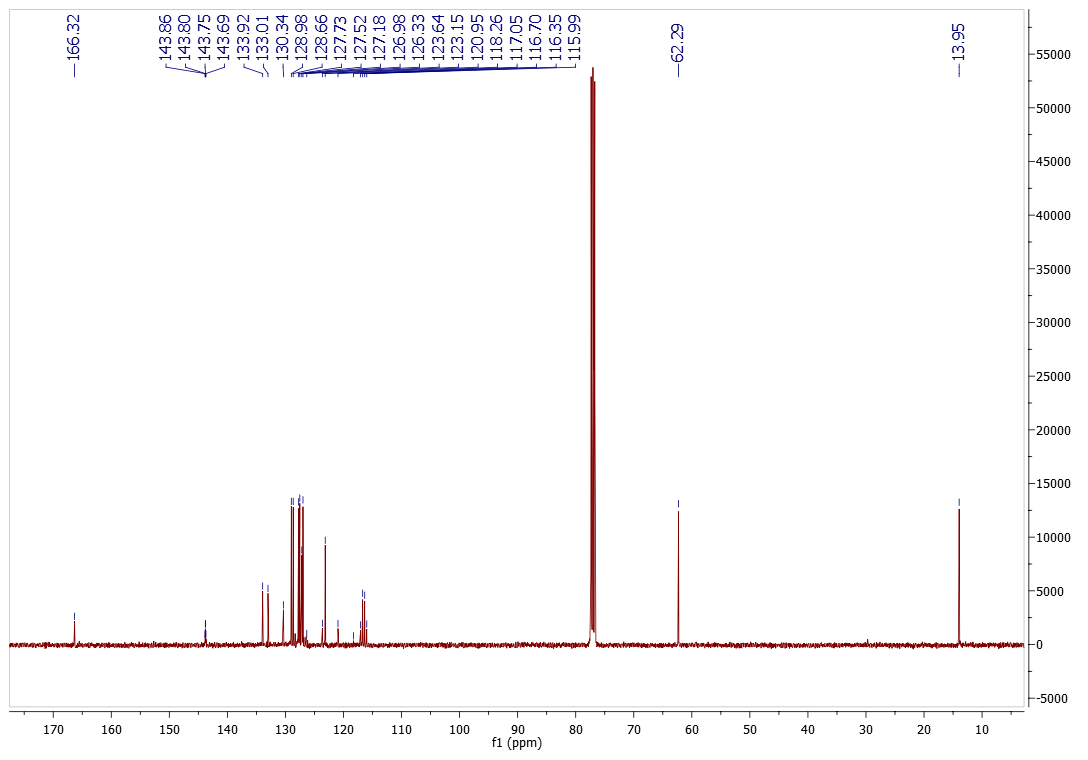


*Ethyl (E)-3-(naphthalen-2-yl)-3-(4-nitrophenyl)acrylate (****28****).*


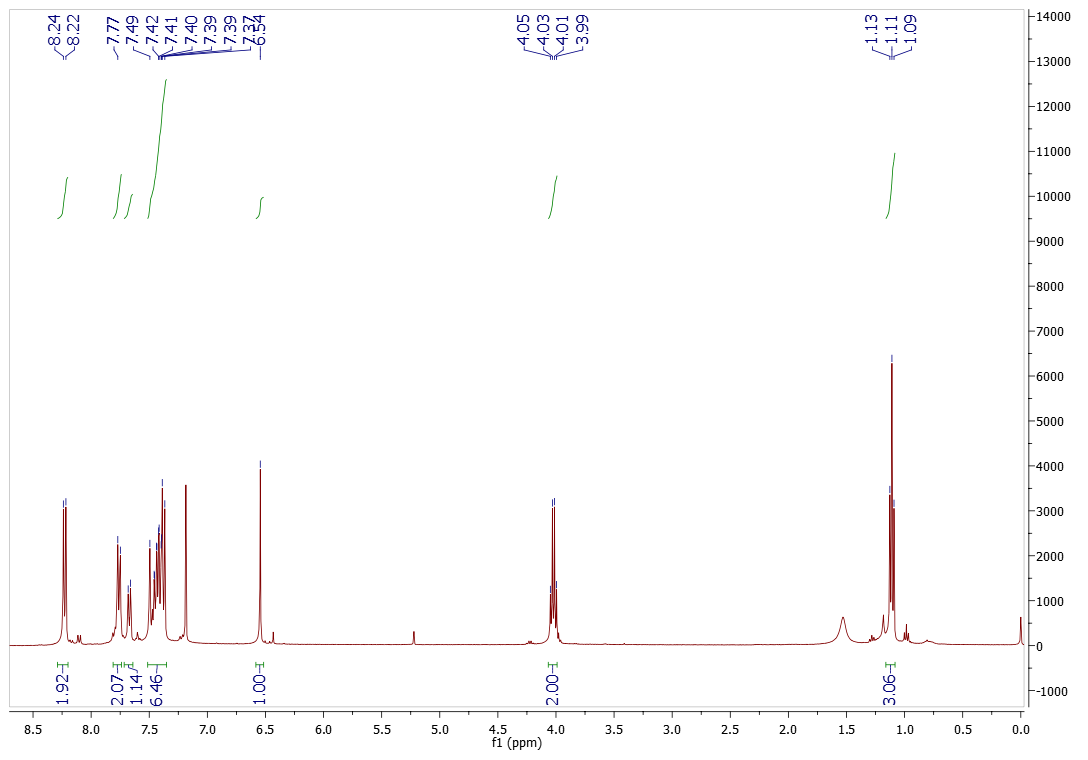


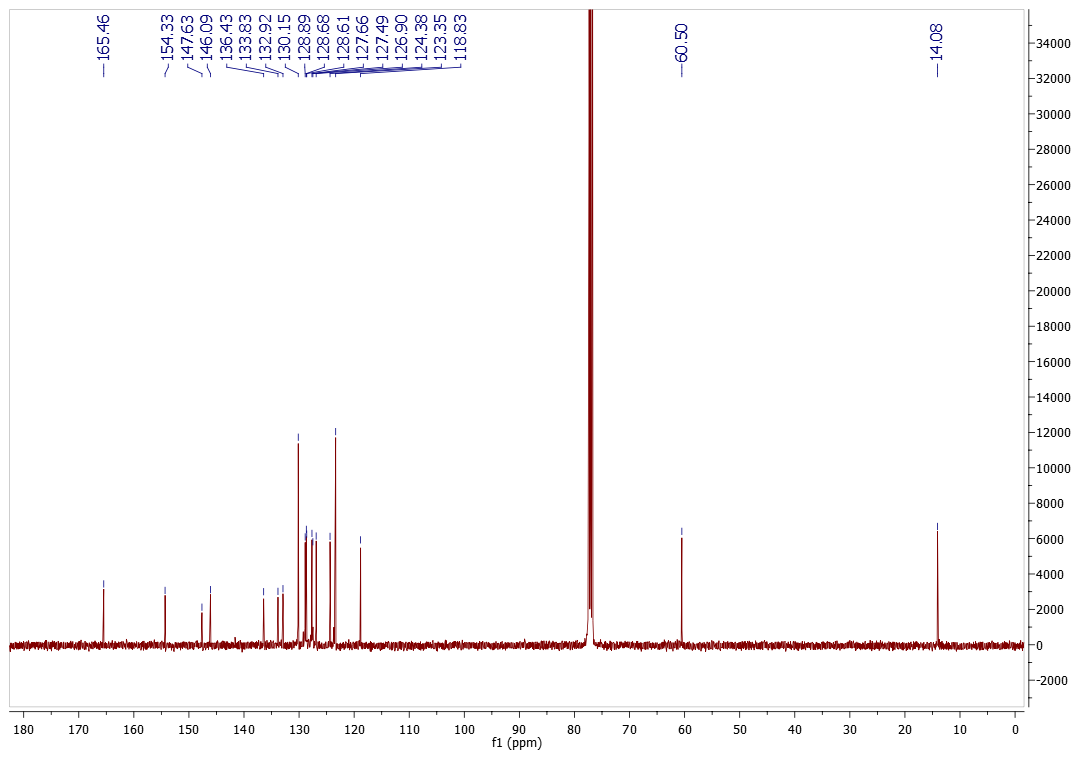

Supplement: Supplementary file 1 [file DataSheet1.docx]
